# Immune and biochemical responses in skin differ between bovine hosts genetically susceptible and resistant to the cattle tick *Rhipicephalus microplus*

**DOI:** 10.1186/s13071-016-1945-z

**Published:** 2017-01-31

**Authors:** Alessandra Mara Franzin, Sandra Regina Maruyama, Gustavo Rocha Garcia, Rosane Pereira Oliveira, José Marcos Chaves Ribeiro, Richard Bishop, Antônio Augusto Mendes Maia, Daniela Dantas Moré, Beatriz Rossetti Ferreira, Isabel Kinney Ferreira de Miranda Santos

**Affiliations:** 1 Departament of Biochemistry and Immunology, Ribeirão Preto School of Medicine, University of São Paulo, Ribeirão Preto, SP 14049-900 Brazil; 2 Department of Animal Sciences, University of Illinois at Urbana-Champaign, Urbana, IL 61801 USA; 3 Laboratory of Malaria and Vector Research, National Institute of Allergy and Infectious Diseases, National Institutes of Health, Bethesda, MD USA; 4 International Livestock Research Institute, Nairobi, Kenya; 5 Department of Basic Sciences, School of Animal Science and Food Technology, University of São Paulo, Pirassununga, SP 13635-900 Brazil; 6 Ribeirão Preto School of Nursing, University of São Paulo, Ribeirão Preto, SP Brazil; 7 Department of Genetics and Evolution, Federal University of São Carlos, São Carlos, SP 13565-905 Brazil; 8 Integrative Medicine Program, School of Medicine, University of California Davis, Sacramento, CA 95817 USA; 9 Embrapa Pecuária Sudeste, São Carlos, SP 13560-970 Brazil; 10 Department of Veterinary Microbiology & Pathology, Washington State University, Pullman, WA 99164-7040 USA

**Keywords:** Transcriptome, Differentially expressed genes, *Bos indicus*, *Bos taurus*, Skin, *Rhipicephalus microplus*, Salivary glands

## Abstract

**Background:**

Ticks attach to and penetrate their hosts’ skin and inactivate multiple components of host responses in order to acquire a blood meal. Infestation loads with the cattle tick, *Rhipicephalus microplus,* are heritable: some breeds carry high loads of reproductively successful ticks, whereas in others, few ticks feed and reproduce efficiently.

**Methods:**

In order to elucidate the mechanisms that result in the different outcomes of infestations with cattle ticks, we examined global gene expression and inflammation induced by tick bites in skins from one resistant and one susceptible breed of cattle that underwent primary infestations with larvae and nymphs of *R. microplus*. We also examined the expression profiles of genes encoding secreted tick proteins that mediate parasitism in larvae and nymphs feeding on these breeds.

**Results:**

Functional analyses of differentially expressed genes in the skin suggest that allergic contact-like dermatitis develops with ensuing production of IL-6, CXCL-8 and CCL-2 and is sustained by HMGB1, ISG15 and PKR, leading to expression of pro-inflammatory chemokines and cytokines that recruit granulocytes and T lymphocytes. Importantly, this response is delayed in susceptible hosts. Histopathological analyses of infested skins showed inflammatory reactions surrounding tick cement cones that enable attachment in both breeds, but in genetically tick-resistant bovines they destabilized the cone. The transcription data provided insights into tick-mediated activation of basophils, which have previously been shown to be a key to host resistance in model systems. Skin from tick-susceptible bovines expressed more transcripts encoding enzymes that detoxify tissues. Interestingly, these enzymes also produce volatile odoriferous compounds and, accordingly, skin rubbings from tick-susceptible bovines attracted significantly more tick larvae than rubbings from resistant hosts. Moreover, transcripts encoding secreted modulatory molecules by the tick were significantly more abundant in larval and in nymphal salivary glands from ticks feeding on susceptible bovines.

**Conclusions:**

Compared with tick-susceptible hosts, genes encoding enzymes producing volatile compounds exhibit significantly lower expression in resistant hosts, which may render them less attractive to larvae; resistant hosts expose ticks to an earlier inflammatory response, which in ticks is associated with significantly lower expression of genes encoding salivary proteins that suppress host immunity, inflammation and coagulation.

**Electronic supplementary material:**

The online version of this article (doi:10.1186/s13071-016-1945-z) contains supplementary material, which is available to authorized users.

## Background

The skin is the largest organ of vertebrates and the target for infestation and feeding by over 15,000 species of hematophagous arthropods. Haematophagy evolved at least 300 million years ago in the Devonian Period and resulted in modifications in the composition of arthropod saliva, which acquired inhibitors of vertebrate defense responses [1]. Due to advances in genomics, the different strategies for haematophagy and the pharmacological repertoires of arthropod saliva are now much better defined [2, 3]. However, with the possible exception of mites and their role in atopic dermatitis [4–6], very little is known about host cutaneous reactions to ectoparasites and defense strategies for eliminating them. Since haematophagous ectoparasites pose a formidable selective pressure, the study of arthropod-infested skin can reveal if there are specialized defense mechanisms to control these ectoparasites. Furthermore, knowledge about local reactions to bites and arthropod saliva has become crucial for the development of vaccines against vector-borne diseases because salivary antigens of vectors can be important components of vaccines against vector-borne diseases [7, 8]. Indeed, immunity to salivary proteins of various vectors affects the outcome of infections with the causative agent of diseases that they transmit [9, 10]. A potential etiological link exists between autoimmune diseases of the skin and previous exposure to salivary antigens of haematophagous insects [11].

Hard ticks are vectors of many diseases of great importance for public health and livestock production. As long-term feeders, they are in contact with components of host defenses for relatively long periods that range from days to weeks. In order to create a hemorrhagic feeding pool of blood in their host’s skin, ticks use their mouthparts to tear the epidermis and dermis into which they spit components of their saliva that destroy the skin extracellular matrix, neutralize many defense responses such as coagulation, inflammation and wound repair [3] and build an attachment scaffold of cement. The tick *Rhipicephalus microplus* specializes on cattle and other large bovids, however success of its blood-feeding depends on the breed of the bovine host. Taurine breeds suffer debilitating infestations with hundreds of feeding parasites, whereas indicine breeds typically exhibit few engorging females that lay smaller batches of eggs than females fed on susceptible hosts. These contrasting tick burdens are highly heritable [12, 13] and offer a useful model to study the mechanisms that result in resistance to blood-feeding ectoparasites. At the same time, different levels of host immunity may affect the composition of tick saliva, contributing to these outcomes.

In order to gain insights into the different host defense mechanisms that control hematophagous ectoparasites and result in different tick loads we addressed the following hypotheses: (i) tick bites induce changes in gene expression profiles in the skin of their hosts that will highlight the proteins and defense pathways that participate in skin reactions to ticks; (ii) relative to skin from animals of a tick-susceptible breed of cattle, skin from animals of a tick-resistant breed provide baseline and reactive expression profiles of genes that will indicate the proteins and defense pathways involved in repelling and/or expelling ticks more efficiently from the host’s skin; (iii) differences in the local reaction to bites in resitant and susceptible hosts will affect expression of genes encoding secreted salivary proteins of the tick that mediate parasitism.

Herein, we describe and compare the transcriptional and corresponding inflammatory response profiles in cutaneous reactions to tick bites elicited by the first two developmental stages of *R. microplus*, larvae and nymphs. We examined these reactions in an indicine and a taurine breed of cattle that present contrasting phenotypes of infestation, Nelore and Holstein, respectively resistant and susceptible to tick infestations as ascertained by the number of ticks and the reproductive success of female ticks completing their life-cycles on these two types of host. We also examined if different levels of host immunity affect the transcriptional profiles in the feeding parasite’s salivary glands and how expression of these tick genes correlated with gene expression and inflammation in skin of the two types of bovine breeds.

## Methods

### Hosts, phenotypes of infestations and sampling of skin

The skin biopsies were collected from approximately 6-month old calves, four of the Nelore breed (genetically tick-resistant, *Bos taurus indicus*), and four of the Holstein breed (genetically tick-susceptible, *Bos taurus taurus*). The calves were maintained free of ticks using the following measures: the pregnant mothers were strategically treated with acaricides and maintained in a clean pasture; the newborn calves were housed in sand hutches during the weaning period and were subjected to strategic acaricide treatments. Before tick infestation, biopsies of skin were collected from all animals to provide baseline data. Calves were then infested artificially with 10,000 15-day-old unfed larvae from our colony maintained on Holstein oxen during the parasitic stage. Skin biopsies were collected from infested animals on the second and ninth days after larvae were released. On the second day after infestation two types of skin samples were collected: one was directly associated with feeding larvae and one did not contain a feeding tick, but was designed to monitor potential systemic stress responses of the skin to the infestation. Skin obtained on the ninth day contained the reactions to feeding nymphs (see the experimental design in Additional file 1: Figure S1a). All biopsies were obtained with disposable punches 6 mm in diameter. This diameter was designed to encompass the area of inflammation of tick-bitten skin in order to avoid mixing inflamed with non-inflamed tissues and consequent mixing of different profiles of RNA transcripts (Additional file 2: Figure S2). The phenotypes of the two breeds for resistance and susceptibility to ticks were confirmed by counting female ticks > 4 mm in length, on the left side of each animal on the 21st day after release of larvae. Holsteins, the tick-susceptible breed,exhibited a significantly (*t* test, t(5.254) df = 6 *P* = 0.019) larger number of engorging females (597.0 ± 208.0 ticks per animal) than Nelores, the the tick-resistant breed (42.5 ± 35.8 ticks per animal, Additional file 3: Table S1), as expected [12, 13]. The institutional Animal Ethics Committee of the University of São Paulo approved the experiments described in this work.

### Histology and immunohistochemistry of skin

For histological procedures, skin biopsies placed directly into buffered formalin (pH 7.0) fixative were embedded in paraffin and 4–5 μm thick sections were made. The skin sections were stained with Hematoxilin and Eosin for histophatological analysis and total cells counts, and May Grünwald-Giemsa for differential cell counts. For immunohistochemistry procedures, skin biopsies placed directly into optimum cutting temperature medium (Sakura Finetek), snap‐frozen in liquid nitrogen, and stored at -196 °C until analysis respectively, at -80 °C until processing. Four-five μm thick cryostat sections were dried on glass slides (Star Frost, Mercedes Medical, FL, USA) and fixed in cold acetone. Sections were incubated with PBS/milk 5% plus anti-goat Ig (1:100) for 30 min, followed by 2 h incubation (37 °C) with mouse IgG1 anti-bovine CD3 (1:100) (VMRD, Pullman, WA, USA), mouse IgG1 anti-bovine CD21 (1:100) (AbD Serotec BioRad, Hercules, CA, USA), and mouse IgG2a anti-bovine WC1 (1:100) (AbD Serotec BioRad). After washing with PBS, sections were incubated for 30 min with biotin-labeled goat IgG anti-mouse Fc (1:500) (Santa Cruz, CA, USA). They were washed three times, incubated with avidin-biotin-peroxidase complex, the color developed with 3,3’-diaminobenzidine (Vector Laboratories, Burlingame, ON, Canada) and counterstained with May Grünwald-Giemsa. Tick-infested sections of skin were categorized into three zones according to distance from tick attachment site, only cells in zones one and two were counted (Additional file 2: Figure S2). The counting areas were limited by a Reichart integrating graticle (Austria/PK6, 3× mm) adapted to a microscope (Olympus, Tokyo, Japan) for analysis performed under light microscopy (objectives 40× and 100×). Cells from areas of 0.0625 mm^2^ into the dermis were counted and the means of each area were used for further analyses.

### Isolation of RNA from skin

For isolation of total RNA, skin biopsies were placed in RNALater solution (Ambion, Austin, Texas, USA) and stored at -80 °C until processing. Skin biopsies were removed from RNALater solution, wrapped in heavy-duty aluminium foil and snap-frozen in liquid nitrogen before being pulverized on a liquid nitrogen cooled-metal block (Biospec, OK, USA) with a hammer. The pulverized tissue was placed in a 2 ml screw cap tube containing 1 ml of Trizol reagent (Invitrogen, Life Technologies Corporation, CA, USA). The tissue was spun at 12,000× *g*, for 10 min at 4 °C, to remove extracellular membranes and other insoluble material. Total RNA extraction was then performed with the SV Total RNA Isolation System kit (Promega Corporation, Madison, WI, USA) according to the manufacturer’s instructions and stored at -80 °C until use. The RNA samples were quantification by Nanodrop capillary spectrophotometer (Thermo Fisher Scientific Inc., MA, USA). The integrity and purity of isolated RNA were determined by the “Lab on a Chip” method using Bioanalyzer 2100 (Agilent Technologies, Palo Alto, USA) following the manufacturers’ instructions. The analyzer allows for visual examination of both the 18S and 28S rRNA bands as a measure of RNA integrity numbers (RIN) ranged from 7.1 to 9.1.

### Affymetrix GeneChip gene expression analysis

Total RNA (150 ng) was used to synthesize double-stranded cDNA using the One-Cycle cDNA Synthesis Kit (Affymetrix, Santa Clara, CA, USA). The cDNA served as a template to synthesize biotin-labeled antisense cRNA using an IVT Labeling Kit (Affymetrix). Fifteen μg of labeled cRNA was fragmented and hybridized to the Affymetrix GeneChip® Bovine Genome Array (containing 23,000 transcripts, representing over 19,000 UniGene clusters) as described in the Affymetrix GeneChip® protocol (Affymetrix). Chip hybridization, washing, and staining were performed according to the Affymetrix recommended protocols. After scanning, the digitalized image data were processed using Affymetrix GeneChip® Operating Software (GCOS) and initial analysis was performed using the same software to assess array quality. Signal intensities for each gene were obtained using the robust multiarray average (RMA) function of the Affymetrix package in bioconductor (http://www.bioconductor.org). The ArrayExpress accession number for the microarray data reported in this paper is available. Differentially expressed genes (DEG) were identified using the empirical Bayes method implemented in the Limma package and the RankProd package by the Molecular Core - AFIP facility.

### Data analysis and identification of relevant biological processes by MetaSkin

Transcriptional profile clusterization of bovine skin samples used Non-negative Matrix Factorization [14] and Hierarchical clustering (HCL) [15] algorithms described elsewhere. The clustering analyses were performed with MeV (Multi Experiment Viewer) software [16]. The functional annotation clustering tool was used to cluster gene ontology terms with shared genes into groups to allow an easier functional understanding of the array data.

Genetic pathways were evaluated using the MetaSkin analysis software (Thomson Reuters Systems Biology Solutions). Comparative experimental analysis consisted of mapping gene IDs of the dataset onto IDs in entities of built-in functional ontologies represented in MetaSkin by pathway maps and networks. Statistically significant gene maps were also developed by MetaSkin and represent gene interactions compiled from a curated database of human protein interactions, metabolism, and bioactive compounds.

### Assay for tick behavior

Tick behavior was examined in an arena assay adapted to account for the antigeotropic (i.e. negative gravitropism) behavior of larvae from *R. microplus*. Strips of adhesive tape (3 M, 7 × 1 cm) containing skin rubbings from the groin of resistant and susceptible bovines, from the forehead of human males and control strips that were not contaminated with skin chemistry were fastened to the superior limit of transparent glass containers (20 × 20 × 20 cm) closed with glass lids. Approximately 10,000 unfed larvae ecloded from 500 mg of egg mass two weeks before the experiment were released in the bottom of the boxes and the side containing the strip was photographed with a digital camera (Sony Cyber-Shot W610) at 5, 15 and 30 min after larvae were released. Each tape was fitted in an individual box and the same distance between surface and camera was maintained for photography of all boxes. Density of larvae on strips of tape was quantified by counting ticks with Image J (version 1.47f) freeware. Experiments were performed under natural daylight (12:15 h, T 35 °C; relative humidity (RH) 47%).

### Ticks and dissection of tick salivary glands

Egg masses were oviposited by female *R. microplus* ticks kept in the laboratory, which had been fed previously on Holstein (tick susceptible) and Nelore (tick-resistant) cattle during the parasitic stage. These masses and unfed larvae ecloded from the egg mass were maintained in the laboratory at 27 ± 1 °C, RH ≥ 80% and a 12:12 h photoperiod before being used as described. Unfed larvae (10,000) derived from females fed on tick-susceptible (ULS) or tick-resistant (ULR) hosts were employed to extract RNA 15 days after eclosion. Another group of unfed larvae (ecloded from eggs oviposited by females fed on Holstein) were exposed to host odors by resting them in silk bags (previously washed in double distilled water and air dried) upon the neck of tick-susceptible (ULVS) or tick-resistant (ULVR) hosts for 30 min. The larvae were subsequently deposited in RNALater prior to isolating total RNA. A further group of larvae (10,000) were fed on tick-susceptible (FLS) or tick-resistant (FLR) bovines. After 24 h, the larvae were brushed off the two types of hosts and stored in RNALater. In another set of samples, salivary glands were dissected from nymphs fed on tick-susceptible (SGNS) and tick-resistant (SGNR) hosts. The salivary glands were dissected from these nymphs (30–100 ticks). The dissecting solution was ice cold PBS, pH 7.4. After removal, glands were washed gently in the same ice-cold buffer and stored immediately in RNALater and kept in 70 °C until RNA isolation [17]. The experimental design is depicted in Additional file 1: Figure S1b.

### Preparation of RNA from larvae and nymphal salivary glands

Total RNA was obtained using Trizol reagent (Invitrogen) followed by column-based purification steps with the SV Total RNA Isolation System Kit (Promega Corporation, Madison, WI, USA) following the manufacturer’s protocol. The quantity and quality of the total RNA samples was determined by Nanodrop® and lab-on a-chip analysis using the 2100 Bioanalyzer (Agilent Technologies, Inc., Santa Clara, CA, USA), respectively and the total RNA was used to prepare tick libraries [17].

### Construction of cDNA libraries of tick RNA and sequencing

Non-normalized library preparations for GS FLX titanium (Roche/454 Life Sciences, Branford, CT, USA) sequencing were developed in the High-Throughput Sequencing and Genotyping Unit of the Roy J. Carver Biotechnology Center of the University of Illinois at Urbana-Champaign, based on standard methods used in GS FLX sequencing. Emulsion PCR reactions were performed according to the manufacturer (Roche 454 Life Sciences). Sequencing of the cDNA libraries was performed on a picotitre plate according to the manufacturer’s instructions. Sequencing adapters (A and B) were automatically removed from the reads using signal processing software (Roche 454 Life Sciences). The raw sequence data was deposited in the Sequence Read Archives of the NCBI.

### Bioinformatics tools for annotation of tick transcriptomes

Bioinformatics tools are those described by Garcia et al. [18]. The programs used were written in Visual Basic 6.0 (Microsoft, Redmond, Washington). Bioinformatic analysis and manual annotation were performed for all tick libraries to classify transcripts by families according to their functions. First, the transcripts were classified into Secretory, Housekeeping, Unknown, Transposable Elements and Viral categories. The transcripts in the Secretory category were then re-analyzed and the transcripts embedded within the families of evasins, DAP36 immunossupressant, SAPL15 immunossupressant, chitinases, cysteine proteases with possible basophil activation activity, lipocalins with possible histamine-bining, serotonin-binding and odorant-binding properties, and reprolysin metalloproteases. These embedded transcripts were then analysde, using as selection criterion the presence of the signal peptide indicative of secretion. Following clustering n of the combined data for all of the libraries, we observed that, depending on the library of origin, some clusters of related sequences contained either more or fewer reads than expected from a random distribution. Groups of reads within a family were compared using the Chi-square test in order to verify if there were significant differences in their distribution among developmental stages and origin of the blood meal. Sequence data from ticks used in this work are deposited in GenBank under the transcriptome shotgun annotation portal.

### Statistical analysis

Sigma Stat version 2.03 (SPSS, Chicago, IL) was used to perform the Student’s t-test or Mann-Whitney rank sum test to evaluate significance of differences between among group medians of cell populations in skin from different bovine breeds and to assay for tick behavior. The Chi-square test was used to asses differences between tick Libraries. The *P* value < 0.05 was used to establish the level of significance.

## Results

### Effect of tick bites on the transcriptional profile of host skin from genetically resistant and susceptible hosts

**Fig. 1:**
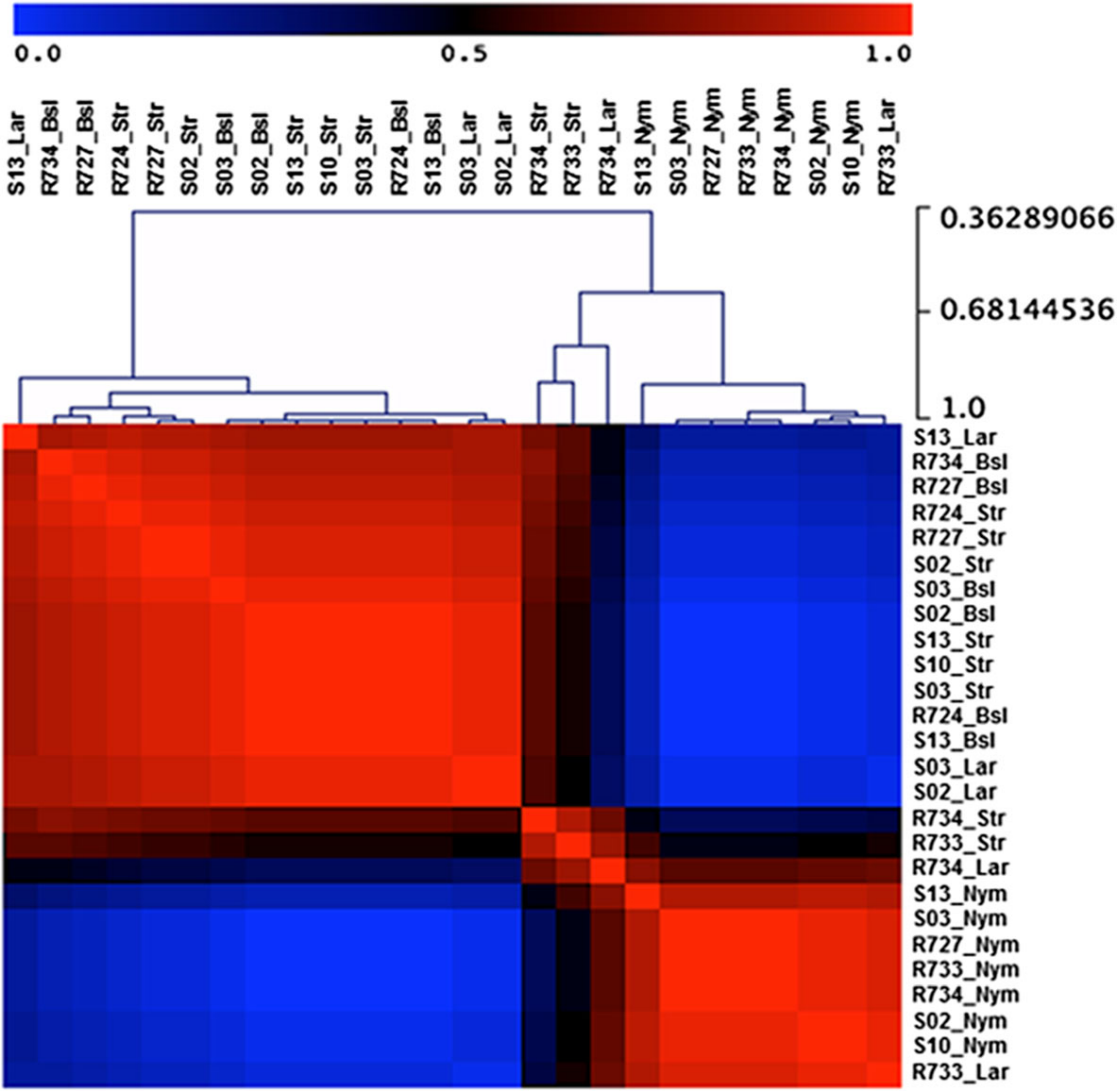
Clustering of all 26 bovine skin samples using Non-negative Matrix Factorization. The data matrix for calculation used intensity values (RMA normalized) for the 1,131 unique differentially expressed genes found across 12 pairwise comparisons (Additional file 5: Table S2). The length of branches in hierarchical tree indicates the degree of similarity between objects (gene expression profiling among the samples) (Cophenetic correlation = 0.86, the highest obtained during analysis), regarding types of experimental groups from which samples are derived and intensity of gene expression. Side scale varying from 0.36 to 1 represents the node height (cluster-to-cluster correlation values). Color bar indicates the degree of correlation where transition from *blue* to *red* means low to high correlation, respectively. *Abbreviations*: S, tick-susceptible hosts; R, tick-resistant hosts; Bsl, baseline skin; Str, stressed skin; Lar, larvae-infested skin; Nym, nymph-infested skin

We first determined the effect of ticks on gene expression profiles in skin from Holstein bulls (see experimental design in Additional file 1: Figure S1a) and Nelore bulls through microarray analysis. Bovine skin was sampled before infestation and on the second and ninth day after larvae were released on the hosts. On the second day, two types of skin were sampled: one contained a larva and, therefore monitored the local reactions to the bite wound and saliva, the second type of sample did not contain a feeding tick, but potentially higlighted systemic stress responses of the skin tissue to the dozens of ticks feeding on the host. Skins obtained on the ninth day profiled host transcriptional responses to feeding nymphs. In total, 12 pairwise comparisons were made to cover multiple aspects of the possible effects of tick infestations on host skin (Additional file 4: Table S2): comparisons were made between the skin samples at different stages of infestation (i.e. baseline, stressed and directly bitten by larvae or nymphs) from the same breed and between skins of the two breeds at the same stage of infestation. Each comparison resulted in a set of DEG, which may exhibit redundancy, i.e. the same gene was found to be differentially expressed in two or more comparisons. To exclude this redundancy, repeated DEG were considered only once and 1,131 unique DEGs was thus identified. To visualize how skin samples are associated, we clustered all skin samples with the Non-negative Matrix Factorization method (NMF, Divergence) based on the signal intensity values normalized by RMA method for unique DEG. We observed two distinct clusters for the 1,131 DEGs (Fig. 1). The first cluster is composed mainly of baseline and stressed skins from both tick-resistant and tick-susceptible bovine hosts, while the second cluster is composed by larval and nymphal-infested skins from tick-resistant hosts and nymphal-infested skins from tick-susceptible hosts. Interestingly, larval-infested skins from tick-susceptible hosts clustered with the tick-resistant hosts’ baseline and stressed skins. This finding suggests that cutaneous responses to ticks develop more gradually in the genetically tick-susceptible Holstein breed, enabling enhanced feeding success to the ticks. Reactions in tick-resistant hosts are similar to those observed in atopic dermatitis, presenting as scaling, weeping and crusty skin, pruritus (characterized by the animals’ attempts to lick and scratch the area) and also dead larvae attached to the same area where larvae were released to infest; similar lesions were not observed in skins from tick-susceptible hosts (data not shown).

**Fig. 2:**
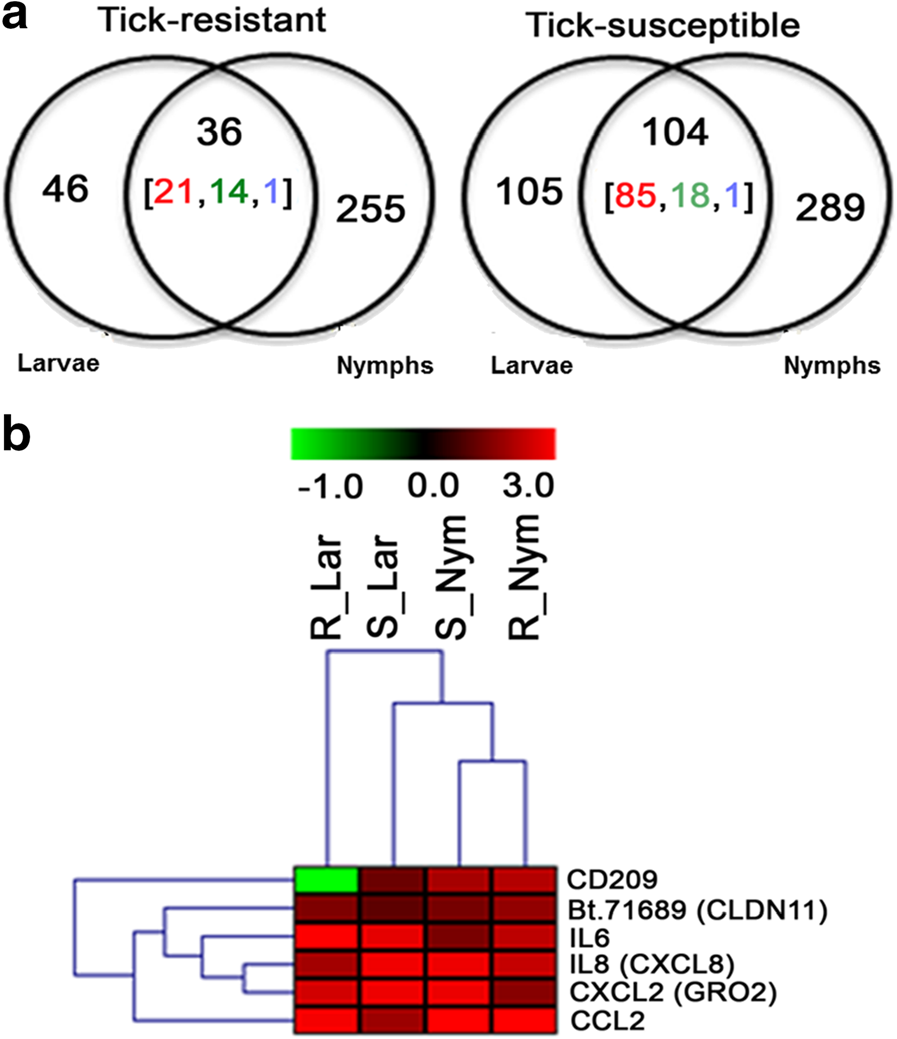
Differentially expressed genes (DEG) found in common across comparisons of tick-infested skin. Genes involved in common in anti-tick responses as well as potential signatures for tick resistance were identified. **a** Venn diagram showing number of DEGs between Str skins versus Lar or Nym skins comparisons within each breed (intra-breed comparison). Numbers inside brackets indicate the number of genes that were upregulated (*red*), downregulated (*green*) or of mixed pattern (*blue*). **b** Hierarchical clustering of expression pattern of six genes found in both overlaps of intra-breed comparisons shown in Fig. 2a. Color bar represent the log2 transformation of fold change values (-1.0 to 3.0) from infested skins when compared to normal skin within same breed; *green* and *red* means downregulated and upregulated genes, respectively. *Abbreviations*: S, tick-susceptible hosts; R, tick-resistant hosts; Bsl, baseline skin skin; Str, stressed skin; Lar, larvae-infested skin; Nym, nymph-infested skin

**Fig. 3:**
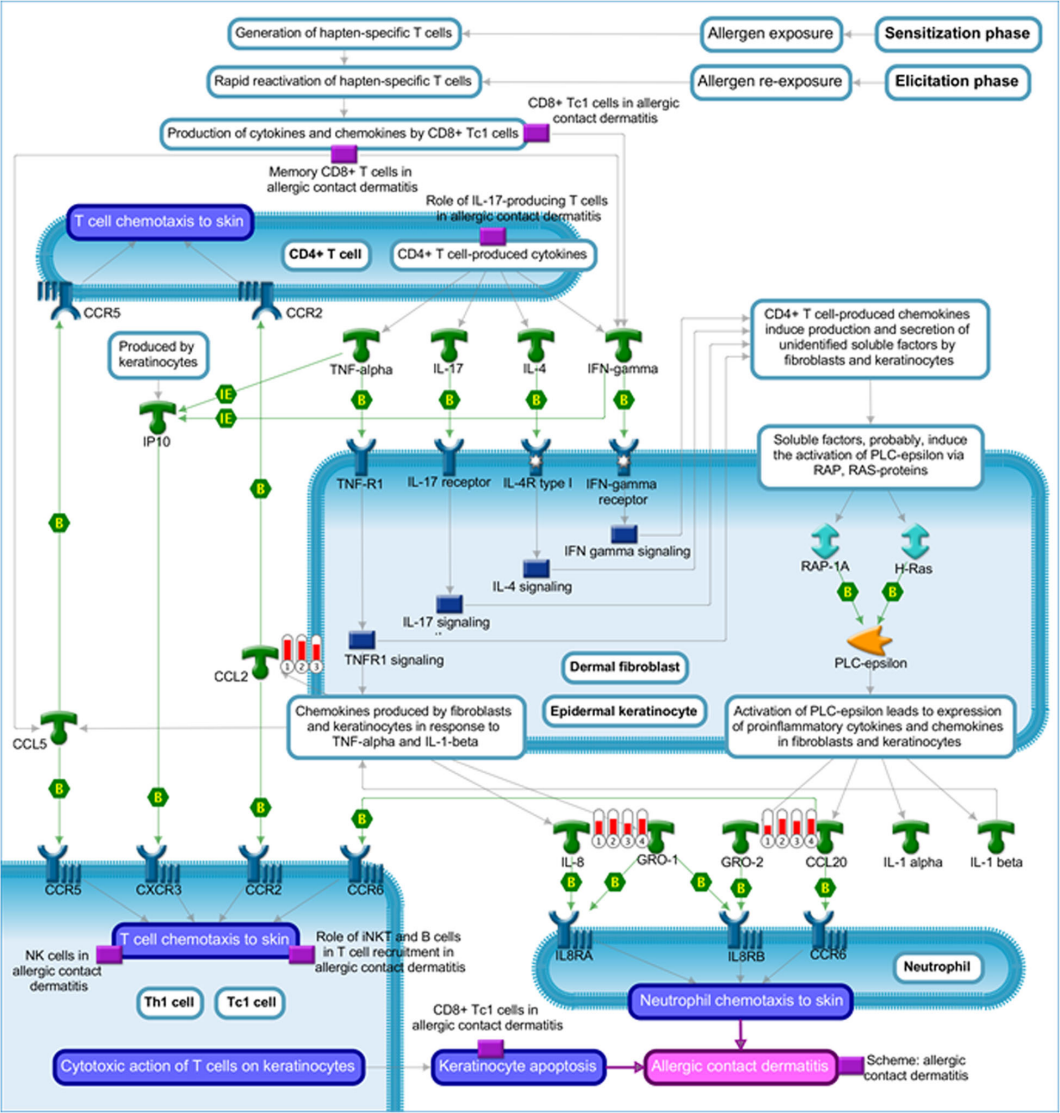
Functional analysis of genes found in common across comparisons of tick-infested skin. The functional analyses were done with MetaCore software (https://portal.genego.com/, Thomson Reuters). The enrichment analyses showed *Role of fibroblast and keratinocytes in the elicitation phase of allergic contact dermatitis* as the most significant pathway map (*P* = 2.095e^-7^, 1.0970e^-5^ FDR). This pathway map showed functional interaction of 3 genes (thermometers) found in both overlaps of intra-breed comparisons shown in Fig. 2b. Colors inside of thermometers indicate that genes were upregulated (*red*) or downregulated (*blue*) in tick-infested skin from both types of hosts. Numbers inside of thermometers indicate the comparisons in which genes are DEG: 1, comparison between stressed and nymph-infested skin from tick-resistant hosts; 2, comparison between stressed and nymph-infested skin from tick-susceptible hosts; 3, comparison between stressed and larvae-infested skin from tick-susceptible hosts; 4, comparison between stressed and larvae-infested skin from tick-resistant hosts

In order to determine the skin responses to tick bites that are common to the two types of hosts (tick-resistant Nelore and tick-susceptible Holstein), we analyzed the DEG resulting from the comparison between stressed and larvae- and nymph-infested skins within each breed using stressed skins as the reference group (values are expressed as the log2 transformation of fold change; Additional file 5: Table S3). The distribution of DEG in Venn diagrams (Fig. 2a) shows that more genes were differentially expressed between both categories of tick-infested skin (i.e. with larvae and nymphs) reative to stressed skin in tick-susceptible hosts than in tick-resistant hosts. Among the DEG common to both larvae- and nymph-infested skin versus stressed skin (36 in tick-resistant hosts and 104 in tick-susceptible hosts; Fig. 2a), six genes, *CXCL2*/*GRO-2*, *CCL2*, *IL8*, *IL6*, *Bt.71689*/*CLDN11* and *CD209*, were found to be common to both breeds, suggesting a common skin reaction to bites of hard ticks. Hierarchical clusterization using the fold change values (log2FC) of these six common DEG (Fig. 2b) showed that all were upregulated in tick-infested skin except for the *CD209* gene, which was downregulated in larvae-infested skin from the tick-resistant hosts (Table 1 and Fig. 2b). The functional analysis of the six genes was performed with MetaSkin software. The enrichment analysis showed that the most significant (*P* = 2.095e^-7^, 1.0970e^-5^ FDR) Pathway Map was that of fibroblasts and keratinocytes in the elicitation phase of allergic contact dermatitis**,** as well as processes associated with the inflammatory response (*P =* 4.44e^-53^), chemotaxis (*P =* 4.28e^-42^), immune response (*P =* 1.41e^-39^), chemokine-mediated signaling pathway (*P =* 4.44e^-32^) and neutrophil chemotaxis (*P =* 3.11e^-25^; Additional file 6: Table S4, 1.1_Pathway_Maps_analysis). This map contained three of the six DEG common to larval- and nymphal-infested skin from tick-resistant and tick-susceptible hosts (*CXCL2*/*GRO-2*, *CCL2* and *IL8*), the signals for which were more robust than the other DEG found to be common to infested skins from these hosts (Fig. 3 and Table 1).

**Table 1:** Differentially expressed genes (DEG) found in common across comparisons between stressed reference skins and larvae- or nymph-infested skins from resistant (R) and susceptible (S) tick hosts. Negative and positive values indicate downregulated and upregulated gene expression in skins containing reactions to bites from larvae or nymphs relative to stressed skin (Additional file 7: Table S4)

Gene symbol	Gene title	Log2 FC				% of identity with human ortholog
		S-Lar^a^	R-Lar^b^	S-Nym^c^	R-Nym^d^	
Bt.71689 (CLDN11)	Claudin-11	1.11	1.50	1.42	1.73	96
CXCL2 (GRO-2)	Chemokine (C-X-C motif) ligand 2	2.57	2.50	2.47	2.12	74
CCL2	Chemokine (C-C motif) ligand 2	1.20	2.90	3.60	3.61	72
IL8 (CXCL-8)	Interleukin 8	2.82	1.94	2.82	2.34	78
IL6	Interleukin 6 (interferon, beta 2)	2.71	2.99	1.44	2.23	51
CD209	CD209 molecule	1.28	-1.05	1.97	1.42	–

*Abbreviations*: *Lar* larvae, *Nym* nymphs, *R* resistant tick host, *S* susceptible tick host, *Str* stressed skin

^a^Comparison of stressed skin with Lar skin from susceptible hosts

^b^Comparison of Str skin with Lar skin from resistant hosts

^c^Comparison of Str skin with Nym skin from susceptible hosts

^d^Comparison of Str skin with Nym skin from resistant hosts

### Impact of host genetic background on transcriptional profile of tick-infested skins

**Fig. 4:**
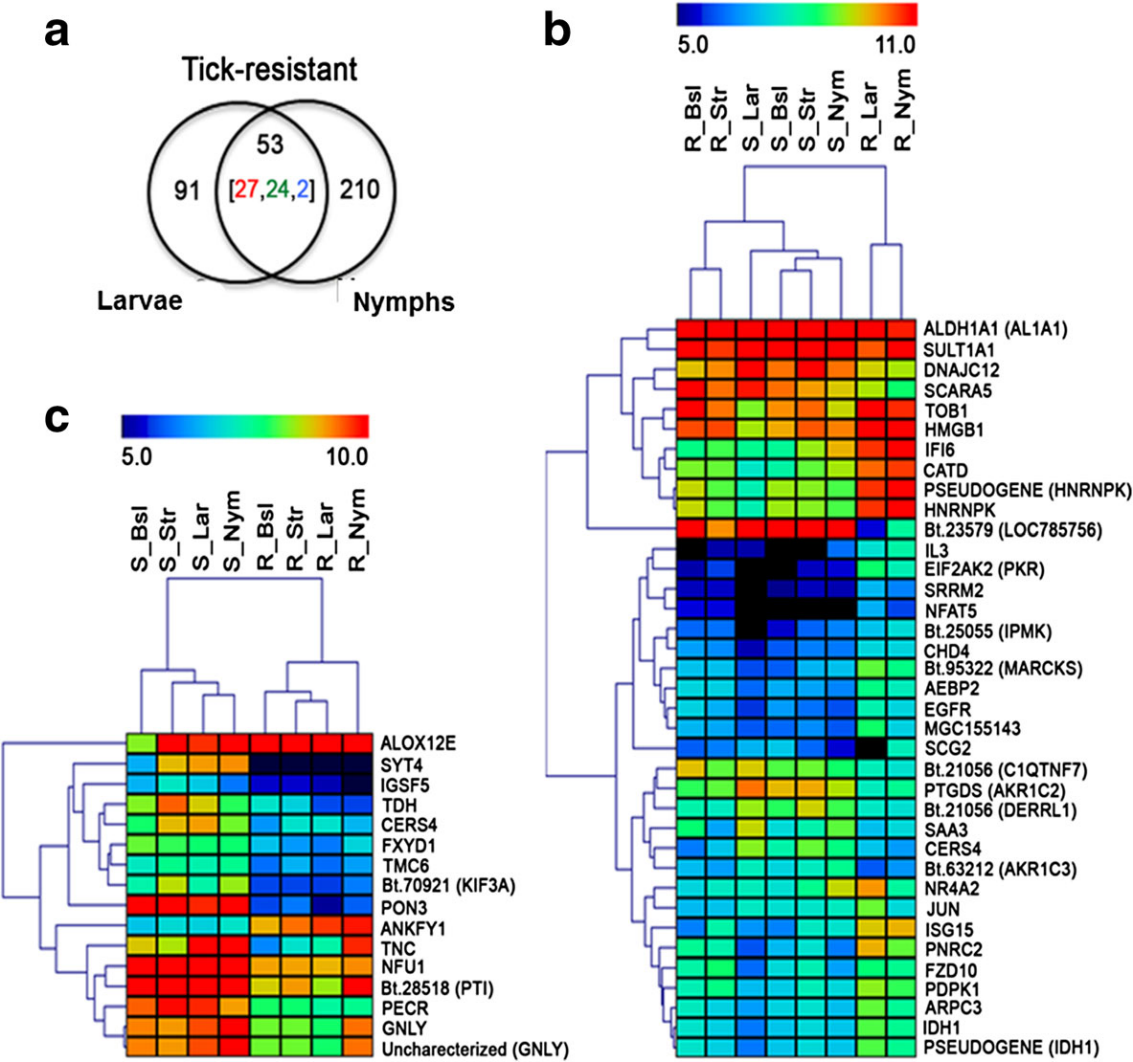
Differentially expressed genes (DEGs) found in common across comparisons between tick-infested skin of tick-resistant and tick-susceptible hosts. Genes involved in common in anti-tick responses as well as potential signatures for tick resistance were identified. **a** Venn diagram showing number of DEGs identified in skins of resistant hosts infested with larvae or nymphs when compared with infested skins of susceptible hosts, (inter-breed comparison). Numbers inside brackets indicate the amount of genes that were upregulated (*red*), downregulated (*green*) or of mixed pattern (*blue*). **b** Hierarchical clustering of 37 DEGs from the 53 DEGs found in common for infested skins in inter-breed comparisons shown in **a** (overlap). Note that the infested skins of resistant hosts are grouped separately (rightmost cluster). **c** Hierarchical clustering of 16 DEGs from the 53 DEGs found in common for infested skins in inter-breed comparisons shown in (**a**) (overlap). The clustering in (**b**) and (**c**) used intensity values (RMA normalized). *Abbreviations*: S, tick-susceptible hosts; R, tick-resistant hosts; Bsl, baseline skin skin; Str, stressed skin; Lar, larvae-infested skin; Nym, nymph-infested skin

In order to determine the effect of the genetic composition of the host on skin responses to tick infestations, we examined which genes were differentially expressed between larval and nymphal-infested skin in both types of hosts (inter-breed comparisons), using skin from susceptible hosts as reference groups (values are expressed in log2 transformation of fold change; Additional file 7: Table S5). The distribution of DEG in Venn diagrams (Fig. 4a) showed that 53 genes were differentially expressed between both larval and nymphal-infested skins of tick-resistant and tick-susceptible hosts. Because they are common to skin reactions against the two developmental stages of the tick, we considered that this set of genes to have altered transcription as a result of tick bites in resistant hosts. However, the 53 DEG common to infested skins do not allow us to discern if this transcriptional modulation is caused only by tick infestations and/or by the host genotype. To ascertain which genes may be influenced by the host genotype, we then searched for DEG in non-infested skins (i.e. baseline skins and stressed skins of tick-resistant and tick-susceptible hosts) that are common to this set of 53 DEGs. We found that 16 genes were also differentially expressed in comparisons of stressed skins from tick-resistant and tick-susceptible hosts. This finding may imply that the transcription of these 16 genes already differs between breeds before infestation, and is an intrinsic feature of the tick-resistant breed. Therefore, of the 53 DEG, 37 were considered to be involved in tick-induced responses mounted by tick-resistant hosts during infestation (Table 2), whereas 16 DEGs were considered be related to innate tick-resistance responses intrinsic to the host genotype (Table 3).

**Table 2:** Differentially expressed genes found in common across comparisons of between skins with tick bites (larvae or nymphs) from resistant and susceptible hosts. Negative and positive values indicate downregulated and upregulated gene expression in skin from tick-resistant hosts as reference groups (Additional file 8: Table S5)

Gene symbol	Gene title	Log2 FC		% of identity with human ortholog
		R-Lar (ref.) X S-Lar^a^	R-Nym (ref.) X S-Nym^b^	
ALDH1A1 (AL1A1)	Aldehyde dehydrogenase 1 family, member A1	-1.59	-1.04	91
SULT1A1	Sulfotransferase family, cytosolic, 1A	-1.62	-1.01	84
DNAJC12	DnaJ (Hsp40) homolog, subfamily C, member 12	-1.78	-0.94	85
SCARA5	Scavenger receptor class A, member 5 (putative)	-1.45	-1.11	89
Bt.23579 LOC785756	Androgen binding protein beta-like	-8.71	-6.33	–
Bt.19274 (C1QTNF7)	C1q and tumor necrosis factor related protein 7	-1.79	-1.00	95
AKR1C2	Aldo-keto reductase family 1, member C2	-2.48	-1.15	72
Bt.21056 (DERL1)	Der1-like domain family, member 3	-0.77	-1.20	83
SAA3	Serum amyloid A 3	-2.40	-1.47	–
CERS4	Ceramide synthase 4	-1.95	-1.71	71
AKR1C3 (Bt.63212)	Aldo-keto reductase family 1, member C3 (Prostaglandin F synthase 1-like)	-1.79	-1.70	78
SCG2	Secretogranin II	-2.58	1.35	87
NR4A2	Nuclear receptor subfamily 4, group A, member 2	2.22	-1.38	100
TOB1	Transducer of ERBB2, 1	2.67	1.17	98
HMGB1	High mobility group box 1	2.09	1.47	98
IFI6	Interferon, alpha-inducible protein 6	2.39	1.14	59
CSTD	Cathepsin D	2.64	1.29	83
Pseudogene (HNRNPK)	Heterogeneous nuclear ribonucleoprotein K	2.71	2.09	100
HNRNPK	Heterogeneous nuclear ribonucleoprotein K	2.71	2.09	100
IL-3	Interleukin 3 (colony-stimulating factor, multiple)	2.32	1.80	29
EIF2AK2 (PKR)	Eukaryotic translation initiation factor 2-alpha kinase 2	3.74	2.34	62
SRRM2	Serine/arginine repetitive matrix 2	1.83	1.10	90
NFAT5	Nuclear factor of activated Tcells 5, tonicity-responsive	2.15	1.35	92
Bt.25055 (IPMK)	Inositol polyphosphate multikinase	2.08	1.09	92
CHD4	Chromodomain helicase DNA binding protein 4	2.07	1.09	99
Bt.95322 (MARCKS)	Myristoylated alanine-rich protein kinase C substrate	–	1.23	92
AEBP2	AE binding protein 2	2.19	1.39	97
EGFR	Epidermal growth factor receptor	2.29	1.49	86
MGC155143	Zinc finger and BTB domain containing 33	2.58	1.35	93
JUN	Jun proto-oncogene	3.16	1.11	98
ISG15	ISG15 ubiquitin-like modifier	2.39	2.21	62
PNRC2	Proline-rich nuclear receptor coactivator 2	3.88	2.61	93
FZD10	Frizzled family receptor 10	2.32	1.51	95
PDPK1	3-phosphoinositide dependent protein kinase-1	2.10	1.32	94
ARPC3	Actin related protein 2/3 complex, subunit 3	2.76	1.13	100
IDH1	Isocitrate dehydrogenase 1 (NADP+), soluble	2.53	1.26	96
Pseudogene IDH1	Isocitrate dehydrogenase 1 (NADP+), soluble	2.53	1.26	96

*Abbreviations: R* resistant hosts and reference skins, *S* susceptible hosts, *Lar* larvae, *Nym* nymphs

^a^Comparison between S-Lar and R-Lar skins

^b^Comparison between S-Nym and R-Nym skins

**Table 3:** Differentially expressed genes found in common across comparisons of between skins without tick bites (baseline or stressed) from resistant and susceptible hosts. Negative and positive values indicate downregulated and upregulated gene expression in skin from tick-resistant hosts as reference groups (Additional file 8: Table S5)

Gene symbol	Gene title	Log2FC		% of identity with human ortholog
		R-Bsl (ref.) X S-Bsl^a^	R-Str (ref.) X S-Str^b^	
ALOX12E	Arachidonate lipoxygenase, epidermal	3.38	2.96	–
SYT4	Synaptotagmin IV	-3.26	–	92
IGSF5	Immunoglobulin superfamily, member 5	-1.20	-1.83	48
TDH	L-threonine dehydrogenase	-1.22	-2.55	–
CERS4	Ceramide synthase 4	-1.63	-1.78	71
FXYD1	FXYD domain containing ion transport regulator 1	-1.56	-1.73	90
TMC6	Transmembrane channel-like 6	-1.28	-1.12	74
Bt.70921 KIF3A	Kinesin family member 3A	-1.79	-2.99	99
PON3	Paraoxonase 3	-4.43	2.96	81
ANKFY1	Ankyrin repeat and FYVE domain containing 1	2.23	2.35	90
TNC	Tenascin C	-2.66	-1.44	86
NFU1	NFU1 iron-sulfur cluster scaffold homolog	-1.08	-1.22	93
Bt.28518	Pancreatic trypsin inhibitor	-1.27	-1.22	–
PECR	Peroxisomal trans-2-enoyl-CoA reductase	-1.50	-1.94	77
GNLY	Granulysin	-1.57	–	37

*Abbreviations: R* resistant hosts, *S* susceptible hosts, *Bsl* baseline, *Str* stressed

^a^Comparison between S-Bsl and R-Bsl skins

^b^Comparison between S-Str and R-Str skins

In order to evaluate the changes in expression of these genes across the different groups of skin types, we performed hierarchical clustering analyses using the intensity values (RMA normalized, Additional file 4: Table S2) for the 37 infestation-induced DEGs (Fig. 4b) and for the 16 DEG associated with the default tick-resistance response (Fig. 4c). The clustering patterns of the skin sample profiles corroborated our classification of gene signatures. As can be observed in Fig. 4b, the larval- and nymphal-infested skins of resistant hosts formed a separate and well-defined cluster (right side) distant from non-infested skins (baseline and stressed skins, left side) reflecting transcriptional modulation of expression of these 37 genes resulting from tick infestation in the resistant breed. The hierarchical clustering of 16 DEGs (Fig. 4c) showed that the groups of bovine skins formed two main groups according to the breed, regardless of whether they were infested or not, confirming that the host genotype exerts a siginificant effect in addition to tick infestation per se upon the transcriptional changes of this set of genes. Note that unbitten skin from resistant, infested animals (stressed skin) and skin from resistant hosts reacting to bites from larvae formed a cluster, displaying a similar transcriptional profile with slight changes from baseline skin from non-infested resistant hosts. This pattern of expression corroborates the idea of a intrinsic tick-resistance response, which becomes increasingly stimulated during infestation (see profile of nymph-infested skin from resistant hosts, Fig. 4c). Furthermore, the cluster analysis showed that gene expression profiles for nymphal-infested skin from resistant and susceptible hosts were clearly different, consistent with the observed phenotypes of tick infestation for the two breeds of hosts (Fig. 4c). The gene encoding epidermal Arachidonate lipoxygenase (*ALOX12E*) was the most upregulated DEG in baseline skin from resistant hosts, with 3.38 log2 transformation of fold change relative to susceptible animals; the gene encoding Synaptotagmin IV (*SYT4*), with -3.26 log2 transformation of fold change, and Paraoxonase 3 (*PON3*), with -4.43 log2 transformation of fold change, were the most obviously downregulated DEG in baseline skin from resistant hosts (Table 3). The MetaSkin analyses did not show any functional interaction between these genes. The first set of genes shown in Fig. 4b is likely involved in mediating resistance responses in cutaneous immunity during infestation, because the expression of most of these genes was significantly upregulated in tick-bitten skins from resistant animals (R-Lar and R-Nym skin samples in Table 2).

**Fig. 5:**
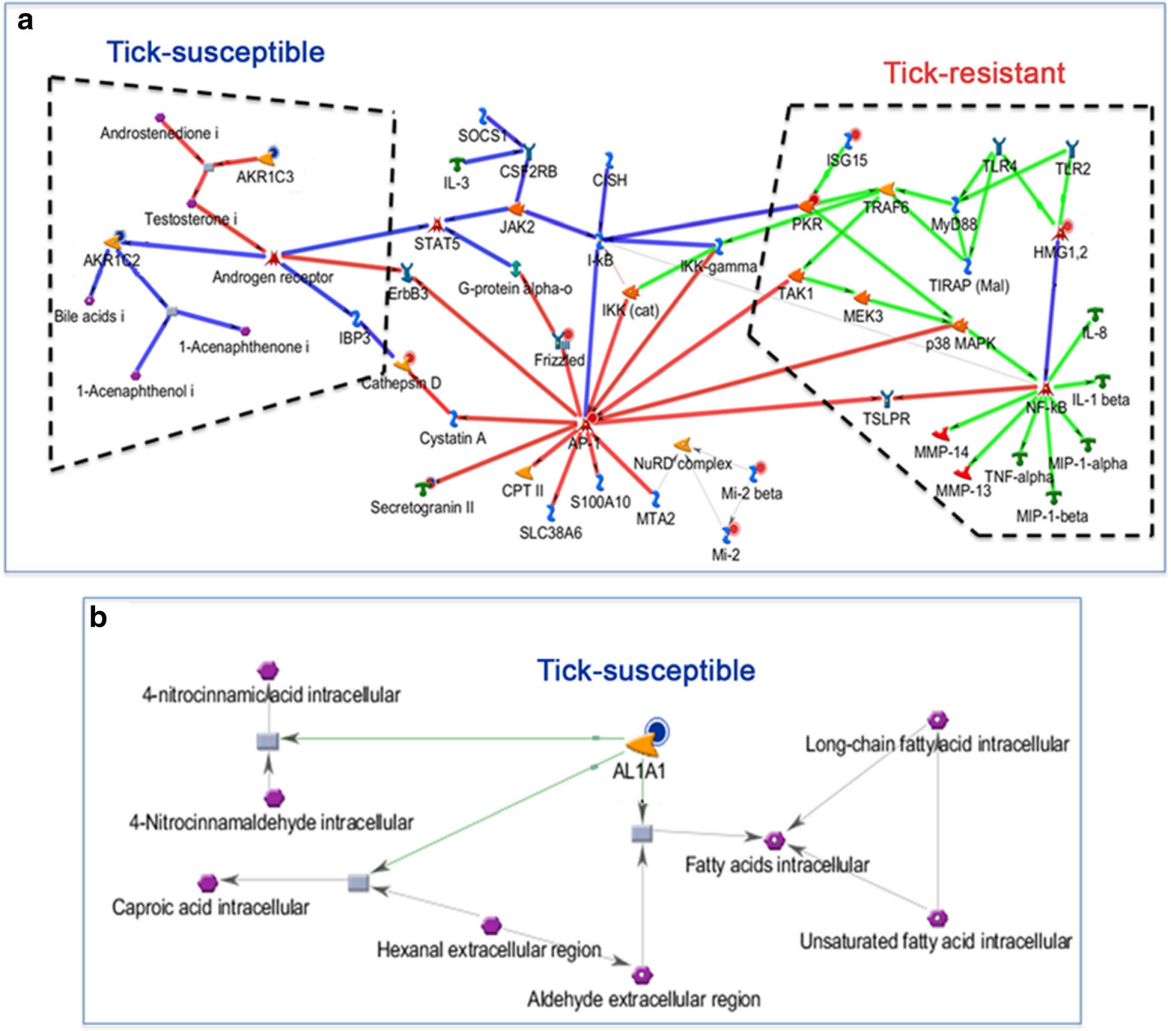
Functional analysis of genes found tick-infested skins in common across comparisons of resistant (R) and susceptible (S) hosts. The functional analyses were done with MetaCore software (https://portal.genego.com/, Thomson Reuters). **a** The DEG were recruited to networks and the three most significant (*P* = 7.44e^-13^, *P* = 1.04e^-15^ and *P =* 7.44e^-13^) in Lar and Nym skins from both types of host (R and S) were merged. The merge shows the functional interactions of the recruited DEG: two DEG (*HGM1*/*HGM*2 and *ISG15*/*PKR*; *red* circles) were upregulated in RLar and RNym relative to reference skins (pathway depicted by *green* lines on the right of Fig. 5a) and two DEG (*AKR1C2* and *AKR1C3*; *blue* circles) were downregulated in RLar and RNym, the same for the pathway depicted by *purple* lines on the left of Fig. 5a). **b** Another DEG was recruited to a significant (*P =* 9.77e^-05^) network in larvae- and nymph-infested skins from both breeds. The network recruited *AL1A1* (*blue* circle), which was downregulated in RLar and RNym relative to reference skins

Among the networks generated by functional analysis of these DEGs, the three most significant (*P* = 7.44e^-13^, *P =* 1.04e^-15^ and *P =* 7.44e^-13^) were the GO processes of the MyD88 dependent toll-like receptor (TLR) signaling pathway, cellular response to organic substances and response to oxygen-containing compounds, as well as the enzyme linked receptor protein signaling pathway and cellular response to growth factor stimulus (Additional file 6: Table S4, 1.2_Network_analysis). These three networks were merged, resulting in two distinct pathways highlighted by boxes of black dotted lines in Fig. 5a: one was composed by DEG upregulated in larvae- and nymph-infested skins of resistant hosts (red circles attached to the DEG, with the network depicted by green lines; box to the right of Fig. 5a) and the other was composed by DEG upregulated in identical comparisons in the skins of susceptible hosts (blue circles attached to the DEG, with the network depicted by purple lines; box to the left of Fig. 5a). The members of the first pathway participate in production of pro-inflammatory cytokines (TNF-α and IL-1β), pro-inflammatory chemokines (MIP1-α or CCL-3 and MIP1-β or CCL-4) and metalloproteases (MMP13 and MMP14), and activation of TLR-2 and/or TLR-4. These cutaneous innate immunity and inflammatory processes are activated directly or indirectly by products of the DEG (*HMG1*,2, *ISG15* and *EIF2AK2*/*PKR*; depicted by red circles, Fig. 5a): the transcription factors HMG1 and HMGB2 (high mobility group box 1 and high mobility group box 2), by ISG15 (Interferon Stimulated Gene 15), a ubiquitin-like protein inducible by IFN-α, -β, and -τ, and by EIF2AK2 (eukaryotic translation initiation factor 2-alpha kinase 2), a protein kinase. These genes are involved in innate immune responses, leading to the sequential nuclear accumulation of NF-kB and expression of attendant pro-inflammatory chemokines and cytokines [19–21]. The second most significant pathway contains the metabolic compounds intracellular testosterone, intracellular bile acids and intracellular 1-acenaphthenone, compounds produced by enzymes in the aldo-keto reductase family 1, members C2 and C3, encoded by the DEG *AKR1C2* and *AKR1C3* (depicted by blue circles; Fig. 5a). Transcription of these genes was downregulated in infested skin of resistant hosts relative to reference skins from susceptible hosts (Table 2).

The functional analyses of DEGs listed in Table 2 revealed another significant (*P* = 9.77e^-05^) network, as well as the GO processes 9‑cis‑retinoic acid biosynthesis, and diterpenoid biosynthesis (Additional file 6: Table S4, 1.3_Network_analysis). The *AL1A1* gene (also called Aldehyde dehydrogenase 1 family, member A1 [*ALDH1A1*], depicted by a blue circle in Fig. 5b, encodes an enzyme that belongs to the aldehyde dehydrogenase family and is directly involved in the production of intracellular fatty acids, intracellular 4-nitrocinnamic acid and intracellular caproic acid (Fig. 5b). *ALA1* presented a high level of expression in all groups of skin, in both tick-resistant and tick-susceptible animals (Fig. 4b), with the exception of tick-infested skin from resistant hosts, where it was a significantly downregulated DEG relative to reference skins (Table 2). The *Bt.23579* gene was strongly downregulated in tick-infested (larvae and nymphs) skin from resistant hosts (Fig. 4b), and was the most significantly downregulated DEG in these types of skin [-8.71 and -6.33 log2 transformation of fold change, respectively (Table 2)]. It encodes androgen-binding protein beta-like (ABPβ-like). The ABPβ-like protein may be involved in the pathway highlighted for tick-susceptible hosts shown in Fig. 5a, because it is an active biological transporter of sex steroids regulating the access of androgens and estrogens to their target tissue and cell types.

### The effect of skin odoriferous stimuli from resistant and susceptible bovines on tick behavior

**Fig. 6:**
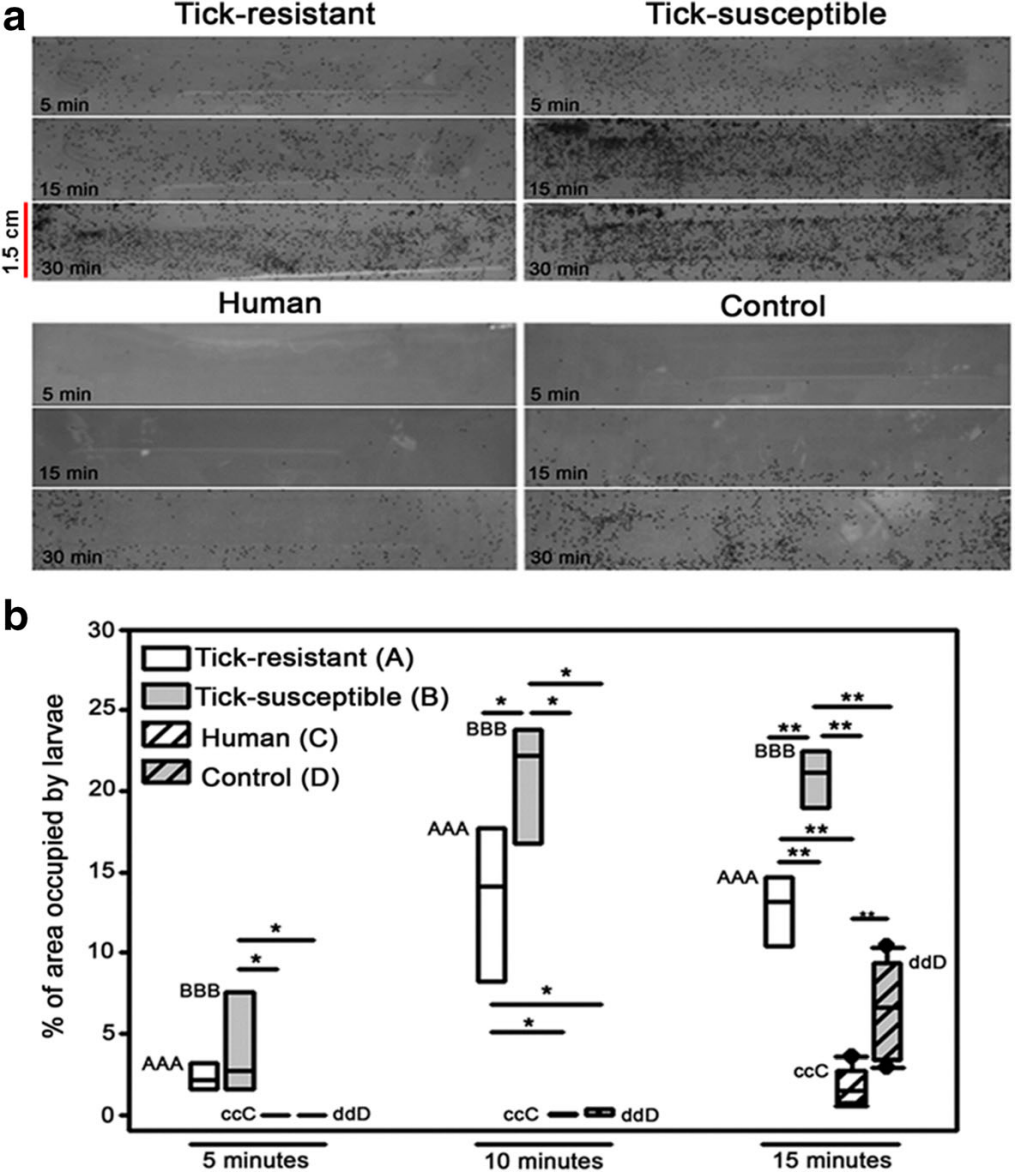
Attraction of skin chemistry from tick-resistant and tick-susceptible hosts for *R. microplus*. **a** Strips (10 × 1.5 cm) of adhesive-backed tape containing rubbings from tick-resistant and tick-susceptible bovine skin, human skin and control tape were fixed on the top of glass containers. Unfed larvae (10,000) were released at the bottom and migration of larvae to the top was registered at 5 min intervals for up to 30 min. The adhesive tapes shown were registered at 5, 15 and 30 min. **b** The percentage of area occupied by larvae recruited to tapes containing skin rubbings from tick-resistant bovine hosts (*white* box plot), tick-susceptible bovine hosts (*grey* box plot), from a human (*dashed white* box plot) and control tape (*dashed grey* box plot) was quantified using Image J (NIH, USA). Values followed by the same capital letter differ significantly (*P* < 0.05) in an intra-group comparison. Asterisks indicate significant differences in comparisons between the different chemistries on the tapes: *P < 0.05; **P < 0.001

Since genes encoding AKR1C2, AKR1C3, AL1A1 and ABPβ-like were among the most prominent DEG and participate in semiochemical communication, we examined if rubbings from skin of tick-resistant and tick-susceptible hosts contained VOC that can be semiochemicals substances affecting tick behavior. Tick behavior was examined in a modified arena assay, which showed that skin VOC differed between the two types of hosts: we show for the first time that compounds from the skin of tick-susceptible bovine hosts are sought for by unfed larvae in significantly larger numbers than compounds from the skin of tick-resistant hosts. Skin rubbings from tick-susceptible hosts attracted significantly more unfed larvae than those from skin of tick-resistant hosts at 10 min (*t *= -2.829, *df* = 9, *P* = 0.020) and 15 min (*t* = 6.184, *df* = 11, *P* < 0.001) after exposure to the rubbings (Fig. 6a). This attraction increased with time indicating that composition of the strips was stable. Both skin extracts attracted significantly more larvae than the control strip (*t *= 4.471, *df* = 14, *P* < 0.001). Interestingly, human skin extracts, included as a control, seemed to repel the ticks since there were significantly less larvae on this strip compared with the control strip at 15 min (Fig. 6b and Additional file 8: Table S6).

### Cutaneous histolopathological features in resistant and susceptible bovines

**Fig. 7:**
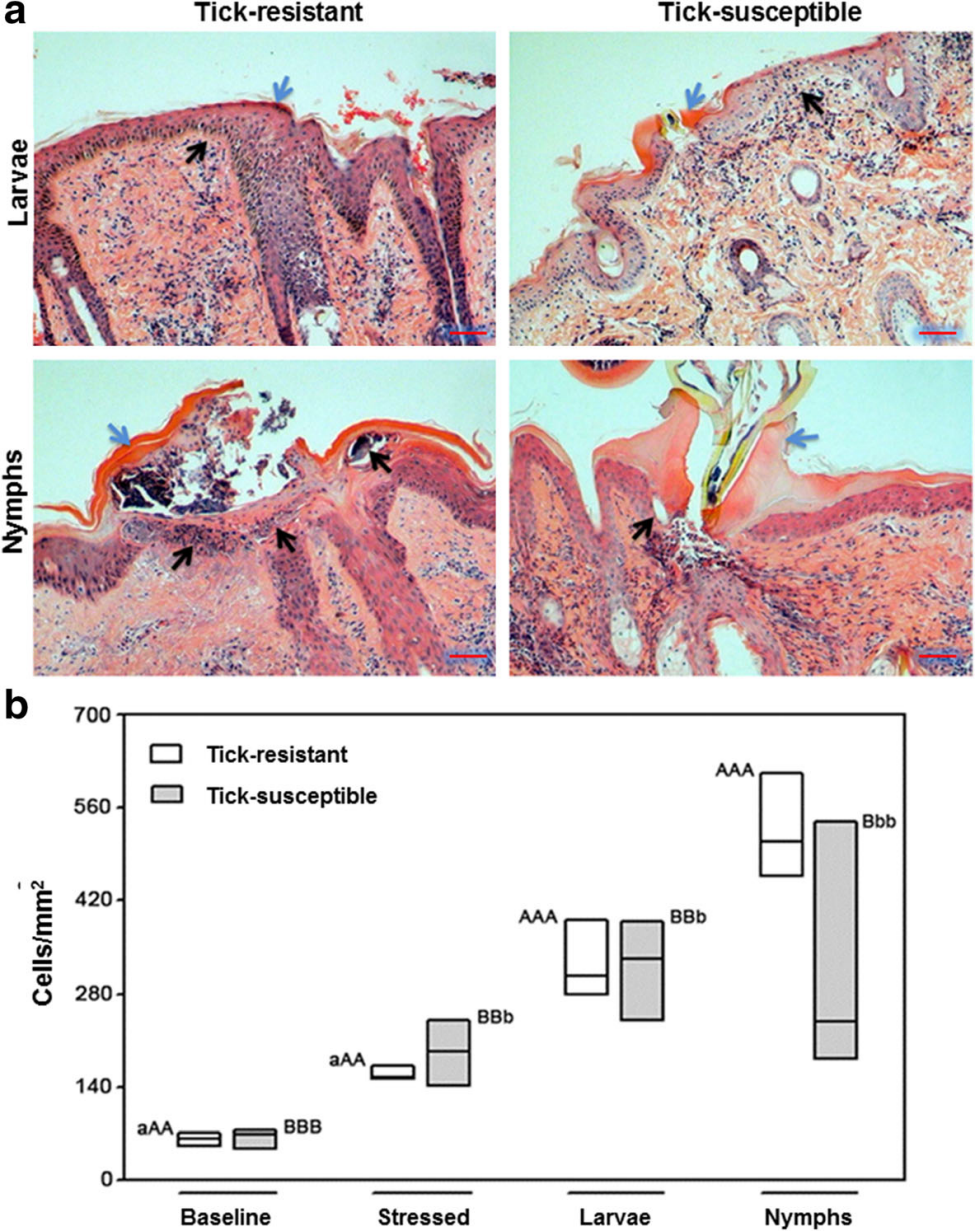
Histopathological analysis of tick-infested skins. **a** Skin biopsies were fixed in formaldehyde and embedded in paraffin. Five micron sections were stained with hematoxylin and eosin. The *blue* arrowheads indicate the cement cone produced by *R. microplus* showing the center of tick lesion (Zones 1 or 2, Additional file 1: Figure S1b), *black* arrowheads indicate the cellular infiltration in the epidermis surrounding cement cone. * Scale-bar*: 50 μm. **b** The inflammatory infiltrating cells were counted into the dermis of tick-resistant (*white* boxes) and tick-susceptible (*gray* boxes) from areas of 0.0625 mm^2^, and the means of each bovine were used from further analyses. Values followed by the same capital letter differ significantly (*P* < 0.05; in intra-breed comparisons). Asterisks indicate significant differences in inter-breed comparisons: *P < 0.05; **P < 0.001

**Fig. 8:**
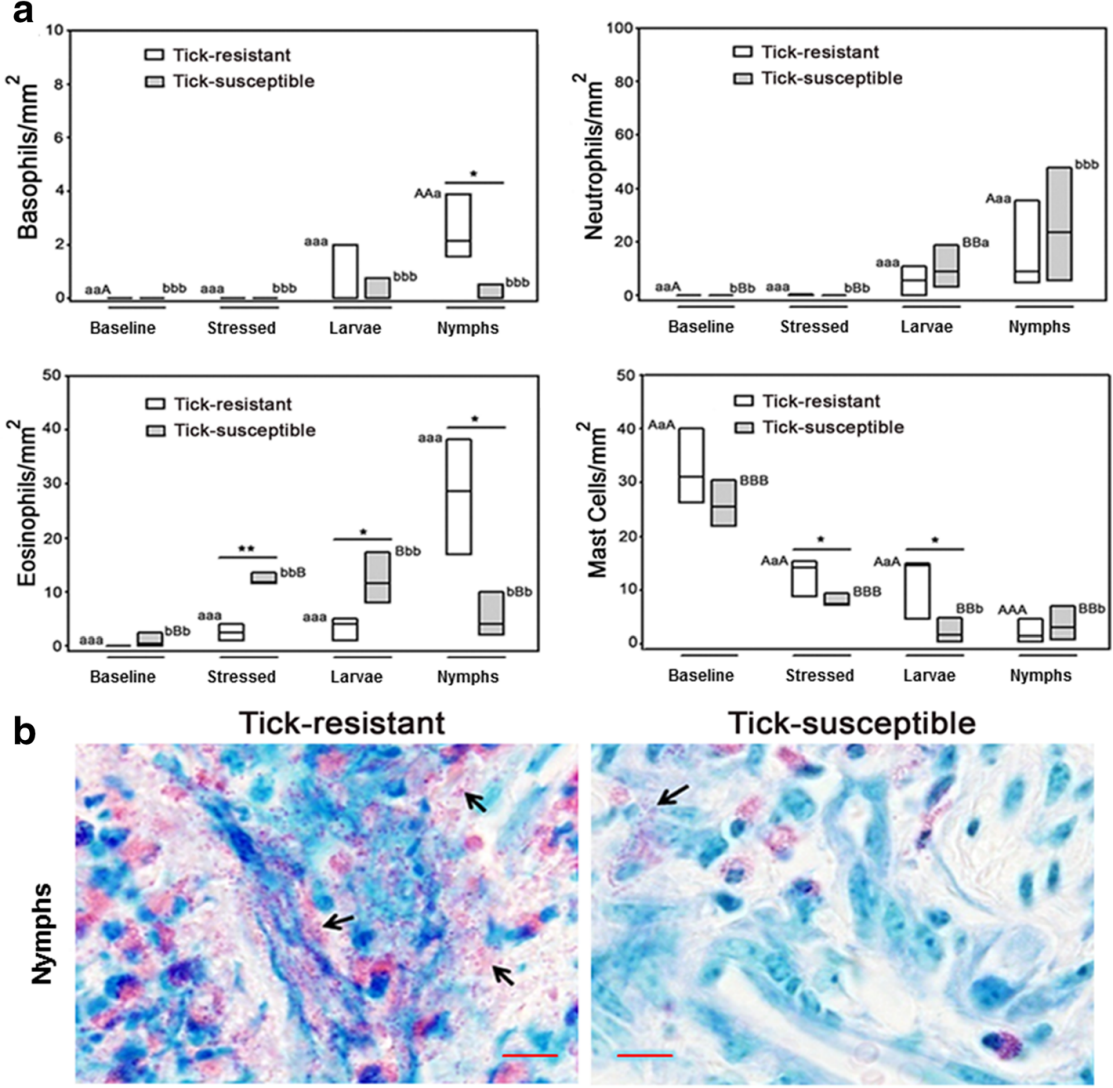
Leukocytes characterization in tick-infested skins. **a** Paraffin-fixed sections were stained with May-Grünwald and Giemsa to differential counts of local granulocytes. The granulocytes were counted into the dermis of tick-resistant (*white* boxes) and tick-susceptible (*gray* boxes) from areas of 0.0625 mm^2^, and the means of each bovine were used for further analyses. Values followed by the same capital letter differ significantly (*P* < 0.05), intra-group comparison. Asterisks indicate significant differences in inter-breed comparisons: *P < 0.05; **P < 0.001; **b** Areas of inflammation in nymph-bitten skin from tick-resistant and tick-susceptible bovines presented conspicuous granules (*black*arrows) densely dispersed in the lesion.* Scale-bar*: 1 μm

We also comprehensively examined histopathological aspects of host responses to ticks and compared the features and the composition of the cellular infiltrate at the feeding sites of larvae and nymphs in the skin of tick-resistant and tick-susceptible hosts. We first assessed general aspects of attachment and feeding (insertion of hypostome and formation of cement cones and feeding pools), which follow a similar pattern in larvae- and nymph-infested skin. The attachment process involved mechanical disruption of the epidermis and deposition of the cement cone through the fissure, bythe mouthparts of ticks (Fig. 7a, blue arrows). In tick-susceptible hosts these processes were uniform, however in resistant hosts they were variable between individual animals and in half of the lesions examined, the presence of tick hypostomes and cement cones was not noted in larval-infested skin. In addition, the epidermal lesions, spongiosis, the formation of microvesicles, vesicles and bullae were more intense in tick-resistant hosts (Fig. 7a, black arrows). Similar lesions have been shown by other studies of reactions to tick infestation in resistant hosts and it has been suggested that such lesions could be responsible for the inability of ticks to engorge normally, or for death at the feeding site [22–24]. The total cell counts of the local cellular infiltrate did not present significant differences between tick-resistant and tick-susceptible hosts, but the number of recruited cells increased significantly as the infestations progressed from the larval to the nymphal stage (Fig. 7b). However, some conspicuous differences were seen between the populations of inflammatory leukocytes recruited by tick-resistant and tick-susceptible hosts (Additional file 9: Table S7). Basophils were recruited in similar, albeit discrete numbers to inflammation elicited by bites of larvae in skins of tick-resistant and tick-susceptible hosts, but nymphally-infested skin of resistant hosts presented significantly (*t* = 3.460, *df* = 4, *P* = 0.026) more basophils than that of nymphally-infested skin of susceptible hosts (Fig. 8a). Eosinophils were recruited in significantly (*t* = -3.825, *df* = 4, *P* = 0.019) larger numbers to larvally -infested skin of susceptible hosts skins as compared to larvally-infested skin of resistant hosts, however this pattern was reversed in nymphally-infested skin of susceptible and resistant hosts, with the differences still being significant (*t *= 3.429, *df* = 4, *P* = 0.027; Fig. 8a). Similar numbers of neutrophils were recruited to inflammation elicited by bites of larvae and nymphs in skins from tick-resistant and tick-susceptible hosts (Fig. 8a). Significantly more mast cells were present in stressed and in larvally-infested skins of resistant hosts than in similar skin samples from susceptible hosts (*t* = -6.909, *df* = 21, *P* < 0.001). Nymphally-infested skins from both types of hosts, susceptible and resistant, contained similar numbers of mast cells, but in resistant hosts the number of mast cells decreased significantly (*t* = -2.884, *df* = 5, *P* = 0.034) in the area of the bite lesion relative to larvally-infested skins of these hosts, suggesting that the mast cells had degranulated (Fig. 8a). Granules were indeed conspicuously and densely dispersed in the area of inflammation of nymph-infested skins of resistant hosts (Fig. 8b).

**Fig. 9:**
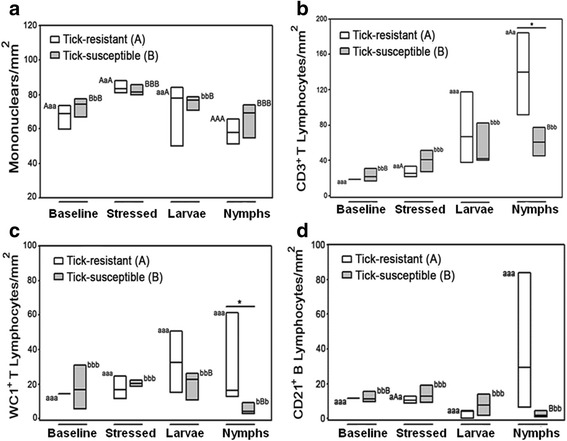
Quantification of mononuclear cells and CD3^+^, WC1^+^ and CD21^+^ lymphocytes in baseline, stressed and tick-bitten skins of tick-resistant (white boxes) and tick-susceptible (gray boxes) bovines. **a** Numbers of mononuclear cells/mm^2^ in paraffin-fixed sections stained with May-Grünwald and Giemsa. **b-d** Numbers of CD3^+^, WC1^+^ and CD21^+^ lymphocytes (Fig. b-d, respectively) in cryopreserved sections stained with peroxidase-immunohistochemistry in a depth of 0.0625 mm^2^ into the dermis. Values followed by the same capital letter differ significantly (*P* < 0.05) between different types of skin from the same breed. Asterisks indicate significant (*P* < 0.05) differences in comparisons between the same types of skin from different breeds

No significant differences were found in the amounts of mononuclear cells present in inflammatory reactions recruited by resistant and susceptible hosts (Fig. 9a), which also presented similar numbers of CD3^+^ T lymphocytes. When ticks reached the nymphal instar, local reactions to the bites recruited significantly (Mann-Whitney U = 0.00, *T* = 26.00, *n*_1_ = *n*_2_ = 4, *P* = 0.029 two-tailed) more CD3^+^ T lymphocytes to the area of inflammation in Resistan hosts relative to susceptible hosts (Fig. 9b). The nymphal stage in this experiment was represented by ticks at the ninth day after infestation, which is approximately the same amount of time an adaptive immune response takes to be mounted by the host. Similar patterns of recruitment were seen for the population of T γδ WC1^+^ lymphocytes, where nymph-bitten skin from resistant hosts contained significantly (Mann-Whitney U = 0.00, *T* = 26.00, *n*_1_ = *n*_2_ = 4, *P* = 0.029 two-tailed) more cells of this population than that of susceptible hosts. This suggests that recognition by T γδ WC1^+^ lymphocytes of molecules s present in tick saliva and/or damaged host tissue is more efficient in resistant bovines (Fig. 9c). B CD21^+^ lymphocytes were also more abundant in nymphally-infested skin of resistant hosts, however, the difference was not significant due to high dispersal of CD21^+^ cells among resistant bovines (Fig. 9d). The distribution of all stained cells is shown in Additional file 10: Figure S3.

The gene expression profiles presented in Figs. 2, 3 and Table 1 demonstrate that expression of genes encoding the neutrophil-recruiting cytokines and chemokines Il-8 or CXCL8 and CXCL2 was significantly upregulated in tick-infested skin relative to unbitten skin from both types of hosts. This is reflected by the composition of cellular infiltrates, which in tick-bitten skin of both types of hosts contained significantly more neutrophils than stressed or baseline skin. On the other hand, relative to stressed skin, expression of the gene encoding the basophil- and T lymphocyte-recruiting chemokine CCL2 was more strongly upregulated only in larvally-infested skin of resistant hosts. This profile is also reflected by the composition of cellular infiltrates, which in nymph-infested skin of resistant hosts presented significantly more CD3^+^ and T γδ WC1^+^ lymphocytes than stressed or baseline skin, recruited by CCL2 produced following gene expression.

### Expression of preditcted inhibitors of host defenses in salivary glands of ticks

We also examined expression profiles of genes encoding secreted immunomodulatory and matrix modulatory proteins in larvae fed on tick-susceptible and tick-resistant hosts and in salivary glands of nymphs fed on tick-susceptible and tick-resistant hosts. We also examined how these profiles correlated with gene expression and with inflammation in the skin of these two types of bovine hosts. We show that the different immune profiles of the hosts significantly affect expression of genes in ticks predicted to encode a class of chemokine-binding proteins known as evasins [25], inhibitors of lymphocyte proliferation and signal transduction known as DAP36 [26] and SALP15 [27], respectively, cysteine proteases and chitinases, lipocalins and matrix-degrading proteases (Table 4). Transcripts for salivary secreted evasins, DAP36 and Salp15 were significantly more abundant in salivary glands of nymphs fed on susceptible hosts. Interestingly, they were not found in larvae, a finding that agrees with the hypothesis that the larval stage does not ingest blood, but instead feeds on tissue exudate. Transcripts for evasins were ten times more abundant in salivary glands of nymphs feeding on susceptible hosts than on resistant hosts. Thus, the ability to evade functions of inflammatory cells is lower in ticks feeding on resistant hosts. Transcripts for matrix-degrading metalloproteases were also more abundant in salivary glands from nymphs feeding on susceptible hosts. Chemokine-binding evasins in the present study were differentially expressed between stressed and bitten skin in both types of hosts (Table 1). Interestingly, all stages of the tick fed on resistant hosts presented significantly more transcripts encoding predicted secreted cysteine proteases, which are known to directly activate basophils [28, 29], than ticks feeding on susceptible hosts. Since intact enzymatic activity is required for these proteases to activate basophils, it is believed that they might cleave a cellular sensor to induce activity [30]. In addition, transcripts encoding secreted chitinases were significantly more abundant in unfed larvae ecloded from eggs oviposited by females fed on resistant hosts, and in salivary glands of nymphs feeding on resistant hosts. Chitin is abundant in the tick’s mouthparts embedded in host skin and tick chitinases may affect availability of this substance, which has also been shown to result in recruitment and activation of basophils [31]. This profile concurs with the finding in this study that inflammation in nymphally-infested skin of resistant hosts contained significantly more basophils than similarly infested skin in susceptible hosts. Published studies indicate that CD4^+^ T cells are necessary for the accumulation of basophils [32, 33]. The present work did not examine CD4^+^ T cells in skin inflammation, but as noted above, it shows that resistant cattle recruit significantly more CD3^+^ T cells to tick bite lesions than do susceptible hosts. Lipocalins were by far the most abundant class of predicted tick secreted proteins and were significantly more highly represented in libraries derived from nymphs feeding on susceptible hosts. Lipocalins bind to small molecules such as histamine, serotonin, leukotrienes and volatile odorants.

**Table 4:** Effect of the origin of blood meal on transcription of genes encoding secreted immunomodulatory and matrix modulatory proteins in the cattle tick *Rhipicephalus microplus*. Non-normalized cDNA libraries were constructed from salivary glands of nymphs fed on tick-susceptible (^a^) or tick-resistant hosts (^b^), from unfed larvae ecloded from eggs oviposited by females fed on tick-susceptible (^c^) or tick-resistant hosts (^d^), from unfed larvae obtained as in (^c^) and exposed to skin volatiles of tick-susceptible hosts (^e^) or tick-resistant hosts (^f^) and from larvae obtained as in (^c^) and fed for 24 on tick-susceptible (^g^) and tick-resistant (^h^) hosts

Functional class of predicted tick secreted protein	Library	Number of reads		Library	Number of reads		
		Observed	Expected		Observed	Expected	*P*-value *χ* ^2^ test
Evasins							
	^a^SGNS	**83**	49	^b^SGNR	6	40	< 0.001
DAP36 immunossupressant family							
	SGNS	**34**	20	SGNR	2	16	< 0.001
SAPL15 immunossupressant family							
	SGNS	**40**	29	SGNR	13	24	0.004
Chitinases							
	^c^ULS	16	25	^d^ULR	**22**	13	0.004
	SGNS	6	13	SGNR	**17**	10	0.010
Cysteine proteases with possible basophil activation activity							
	ULS	7	13	ULR	**13**	7	0.009
	^e^ULVS	221	341	^f^ULVR	**149**	20	< 0.001
	^g^LS	73	92	^h^LR	**38**	20	< 0.001
	SGNS	1	20	SGNR	**35**	16	< 0.001
Lipocalins: Histamine, serotonin, odorant-binding proteins							
	SGNS	**909**	691	SGNR	347	564	< 0.001
Reprolysin metalloproteases							
	SGNS	**217**	124	SGNR	9	102	< 0.001

Values in bold indicate that there were significantly (Chi-square test) more reads in the respective library than in the corresponding library in the comparison

## Discussion

By means of their bites, ticks activate endogenous, host skin-derived inducers of inflammation and, through their secreted salivary gland molecules, they also induce exogenous pathways of inflammation in skin [34]. In this study we sought to understand how the reactions to these challenges result in the contrasting, heritable outcomes of infestations with the cattle tick, *R. microplus*, that occur in indicine and taurine breeds of cattle. We examined global profiles of gene expression in skins from a breed of each type of host before and while undergoing infestations with larvae and nymphs. DEG from all comparisons clustered into hierchies suggesting that cutaneous responses to ticks develop more gradually in tick-susceptible breeds, resulting more effective feeding by the ticks. On the other hand, reactions in resistant hosts were similar to those seen in atopic dermatitis and characterized by the attempts of the parasitized cattle to lick and scratch infested areas.

Several differentially expressed genes were common between the two types of host in both stressed and directly tick-infested skins. Among these were *CXCL2*/*GRO-2*, *CCL2*, *IL8*, *IL6*, *Bt.71689*/*CLDN11* and *CD209*; all were upregulated in tick-infested skin except for the *CD209* gene, which was downregulated in larvally-infested skin from the tick-resistant hosts. This finding suggests a common feature of skin reactions to bites of hard ticks. Functional analyses of these genes indicated that processes involved in allergic contact dermatitis, inflammation and chemotaxis of neutrophils were activated in larvally- and nymphally-infested skin and were compatible with expected responses to bite wounds. The reactions induce a chemokine gradient that recruits neutrophils and T CD4^+^ and CD8^+^ lymphocytes to tick-infested skins from both breeds. T cells, in turn, can produce cytokines (TNF, IL-1, IL-17 and IFN-γ) that stimulate epidermal keratinocytes and dermal fibroblasts to express chemokines assisting recruitment of cells to the skin [35, 36].

IL-6, Claudin-11 and CD209, encoded by the other DEG common to tick-infested skin, can also contribute to local allergic cutaneous inflammation at the site of the tick bite. IL-6 is produced by many cell types including T cells, epidermal keratinocytes and dermal fibroblasts, and is a systemic alarm signal produced by injured tissues, particularly the injured skin [37, 38]. Increased levels of IL-6 have been associated with a number of skin pathologies, such as psoriasis [39], atopic dermatitis [5, 6] and microbial skin infections [40–42]. Of relevance for this study, extracts of mites also induce production of IL-6 by skin fibroblasts [6]. The family of Claudins is formed by at least 23 proteins that are components of tight cell junctions. Distribution of the different Claudin members depends on the tissue and on its physiological state. To date, expression of *Cldn11* and production of Claudin-11 has not been described in skin, however it forms tight junctions between epithelial, endothelial cells and macrophages, moreover expression of *Cldn11* is modulated by IL-6 [43]; increased expression of *Cldn11* may indicate ongoing cellular migration and wound repair at the skin’s hemorrhagic pool where ticks feed.

CD209 (DC-SIGN) is expressed on dermal dendritic-like macrophages and is considered to be a marker of these cells [44], together with the scavenger of haptoglobin-hemoglobin (Hp-Hb) complexes, CD163 [45]. CD209 is a C-type lectin that binds high-mannose glycans on the surface of microbes and of endogenous cells. Its engagement results in production of the anti-inflammatory cytokine IL-10 [46]. Engagement of the Hp–Hb complex by CD163 also mediates anti-inflammatory effects via the release of IL-10 and the induction of heme oxygenase-1 [47], which generates the immunosuppressive metabolites CO, Fe++ and biliverdin from heme. Therefore, this population of macrophages might favor the tick in a *milieu* rich in hemoglobin, which is exactly the environment of the feeding pool. Tick saliva also contains several strategies to target and inhibit functions of dendritic cells [48, 49]. Salp15 is a tick salivary protein that inhibits adaptive immune responses by interacting with CD209 and consequently inhibiting TLR-induced production of pro-inflammatory cytokines by DCs and DC-induced T cell activation by the Raf-1/MEK-dependent signaling pathway [50]. It is noteworthy that expression of CD209 was downregulated in larvally-infested skin from the resistant hosts relative to stressed skin and upregulated in larvally-infested skin of susceptible hosts and in nymphally-infested skin of both host breeds, suggesting that production of anti-inflammatory IL-10 is delayed in tick resistant hosts. Furthermore, our previous work [51] has shown that during exposure of cattle to pastures heavily infested with ticks, levels of Hp increase significantly in susceptible hosts, but not in resistant hosts. Indeed, in tick-susceptible hosts the expression of Hp was significantly higher in larvae-infested skin compared to stressed skin (Additional file 5: Table S3). Collectively, these data suggest that local production of anti-inflammatory molecules may be delayed in resistant hosts relative to susceptible cattle.

We also examined if the genetic composition of the host affected gene expression in baseline and infested skins (inter-breed comparisons, using skins from susceptible hosts as reference groups). Among the DEG observed in these comparisons, we distinguish default genes (i.e. those genes being expressed before ticks are infesting these hosts) and infestation-induced genes. The levels of expression of the 16 “default” genes can be related to innate, tick-resistance responses. Functional analyses of the DEGs indicated that pathways that were differentially recruited in host skin of the two breeds involved the MyD88-dependent toll-like receptor signaling pathway and cellular responses to organic substances, with production of pro-inflammatory cytokines and chemokines, as well as metalloproteases, and activation of toll-like receptors 2 and 4 (TLR-2, TLR-4). These processes are activated directly or indirectly by DEG encoding the transcription factors high mobility group box 1 and 2, interferon-stimulated gene 15 (a ubiquitin-like protein inducible by IFN-α, -β, and -τ), and by eukaryotic translation initiation factor 2-alpha kinase 2. Expression of these three genes was significantly upregulated in larvally- and nymphally-infested skins of resistant host skin samples relative to similar samples from susceptible hosts, and their products lead to the nuclear accumulation of NF-kB and production of attendant pro-inflammatory chemokines and cytokines [19–21]

DEG encoding aldo-keto reductases (*AKR1C2* and *AKR1C3*) and an aldehyde dehydrogenase (*ALA1*) represent additional pathways that may play an important role in the detoxification of tissues from tick saliva and also participate in the metabolism of organic compounds within the skin [52]. In the case of susceptible hosts, theproducts testosterone, bile acids and acenaphthenone may accumulate in their skin; in addition, the enzyme encoded by *ALA1* is important in production of fatty acids that are a key component of the lipid matrix in the outermost layer of the skin This lipid matrix is the source for a range metabolic compounds that feed the skin microbiota [53] and result in the production of volatile odoriferous compounds (VOC), many of which are semiochemicals and may affect tick behavior in the field.

A very strongly differentially regulated gene, the lipocalin ABPβ-like (downregulated in tick-infested skin from resistant hosts), may assist *R. microplus* in the localization of hosts. ABPβ-like is an active biological transporter of sex steroids and regulates the access of androgens and estrogens to their target tissue and cell types. The ABP genes have undergone repeated bursts of gene duplication and adaptive sequence diversification driven by their participation in chemosensation and/or sexual identification [54]. Skin glands are known to be sources of semiochemicals and of olfactory profiles for species-specific vector-host interactions [55, 56]. Skin microbiota, by acting upon androgens [56], also participate in production of body odor. It is noteworthy that testosterone is one of the products of the DEG aldo-keto reductases and thus it is possible that all these DEG may potentially be involved in the production of semiochemical signals for *R. microplus*. Bovine ABP sequences and similar sequences in sheep and goats have been found in skin [54]. Bovine odorant-binding protein (bOBP), an orthologue of ABPβ-like, is a lipocalin that binds 1-octen-3-ol, a VOC of bovine breath and body odor and a potent attractant for blood-feeding vectors like *Anopheles* mosquitos or *Glossina* flies [57]. Interestingly, bOBP was found in milk, urine and plasma of Holstein cattle (the susceptible breed in our model) but not in the sweat of this breed [55]. bOBPs are also known allergens present in bovine skin danders [58]. Moreover, ABPβ-like, which was strongly induced by infestation in susceptible host skins, is a secretoglobin for which physiological functions have not been well defined, but may include tissue repair, immune modulation and mate selection [59]. It is well known that *R. microplus* exhibits a very strong preference for ungulates, especially bovines, but not humans. Overall, our data suggest that one mechanism governing this species-specific, and also breed-specific preference is skin chemistry. If one considers that repellency or attractiveness to hematophagous arthropods is a form of host innate immunity, then odorants generated by the host’s skin chemistry assume an unexpected functional importance.

Different aspects of inflammation of feeding lesions induced by *R. microplus* in skins of resistant and susceptible bovines have previously been investigated by others [22, 60–66], but the present study addressed this topic more comprehensively. We observed a correlation between neutrophil-recruiting chemokines and the composition of cellular infiltrates, skin bitten by ticks from both types of hosts presented significantly more neutrophils than stressed or baseline skin. Expression of the gene encoding the basophil- and T lymphocyte-recruiting chemokine CCL2 was strongly upregulated only in larvally-infested skin of resistant hosts. This profile is also reflected by the composition of cellular infiltrates, which in nymphally-infested skin of resistant hosts presented significantly more CD3^+^ and T γδ WC1^+^ lymphocytes than stressed or baseline skin. Previous studies also found a greater accumulation of CD3^+^ T lymphocytes and WC1^+^ T lymphocytes in the skin of resistant bovines [65, 66], however, among the granulocytes, they did not distinguish between neutrophils, eosinophils and basophils. In the present study, these populations were distinguished and the distribution of the latter two populations differed significantly between resistant and susceptible hosts.

We had previously shown that the genetic background of the host on which cattle ticks feed affects the expression of antihemostatic proteins in tick salivary glands [67]. Therefore, we also examined expression profiles of genes encoding secreted immunomodulatory and matrix modulatory proteins in larvae derived from, exposed to and fed on tick-susceptible and tick-resistant hosts and in salivary glands of nymphs fed on tick-susceptible and tick-resistant hosts. We also examined how these profiles correlated with gene expression and with inflammation in the skin of these two types of bovine hosts. We now show thatthe different immune profiles of the hosts significantly affect expression of genes in ticks predicted to encode chemokine-binding proteins (called evasins) [25], inhibitors of lymphocyte proliferation known as DAP36 [26] and signal transduction SALP15 [27]. Cysteine proteases, chitinases, lipocalins and matrix-degrading proteases were also affected.

Evasins were first described in salivary glands of *Amblyomma variegatum* and *Dermacentor reticulatus* ticks [68] and, among other chemokines, were shown to bind CXCL8 and CCL2, which in the present study were differentially expressed between stressed and bitten skin in both types of hosts (Table 1). DAP36 was first described as an antiproliferative component for lymphocytes in salivary glands of *Dermacentor andersoni* [26], and *Haemaphysalis longicornis* ticks [69]. Salp15, a protein first described in salivary glands of the tick *Ixodes scapularis*, inhibits activation of CD4^+^ T cells and production of IL-2 by binding to CD4 on host T cells [27]. As mentioned above, it also inhibits activation of TLR-dependent pathways by interacting with CD209 [50], a DEG in this study. We have also shown that tick infestations decrease production of saliva-specific antibodies in susceptible hosts, but not in resistant hosts, in spite of the fact that the former receive larger loads of tick saliva [70]. This phenomenon is also possibly mediated by DAP36 and SALP15, which aresignificantly more abundant in ticks feeding on susceptible hosts. Gene duplication is particularly prominent in the evolutionary history of chemokines [71], which are predicted to be more highly duplicated in *B. t. indicus* [72]. Interestingly, evasins, DAP36 and Salp15 were not found in larvae, a finding that agrees with the hypothesis that the larval stage does not ingest blood, but instead feeds on tissue exudate.

Transcripts for matrix-degrading metalloproteases were also more abundant in salivary glands from nymphs feeding on susceptible hosts. Proteases containing a zinc-binding motif common to metalloproteases and similar to the hemorrhagic proteases of snakes were described for the first time in saliva of *I. scapularis* ticks [73] and RNA interference based on metalloprotease-coding sequences prevents ticks from interfering with host fibrinolysis [74]. In the present study they were not found in larvae and were significantly more abundant in salivary glands of nymphs feeding on susceptible hosts than on resistant hosts. Thus, tick reprolysins may inhibit leukocyte adhesion, healing of skin wounds inflicted by tick bites, and may favor creation of the feeding pool.

Interestingly, all stages of the tick fed on resistant hosts expressed significantly more transcripts encoding predicted secreted cysteine proteases, which are known to directly activate basophils [28, 29], than ticks feeding on susceptible hosts. Since intact enzymatic activity is required for these proteases to activate basophils, it is believed that they might cleave a cellular sensor to induce activity [30]. In addition, transcripts encoding secreted chitinases were significantly more abundant in unfed larvae ecloded from eggs oviposited by females fed on resistant hosts and in salivary glands of nymphs feeding on resistant hosts. Chitin is abundant in the tick’s mouthparts embedded in host skin and tick chitinases may affect availability of this substance, which has also been shown to result in recruitment and activation of basophils [31]. This profile concurs with the finding in this study that inflammation in nymph-infested skin of resistant hosts contained significantly more basophils than similarly infested skin in susceptible hosts. Furthermore, CD4^+^ T cells are necessary for the accumulation of basophils [32, 33]. The present work did not specifically examine CD4^+^ T cells in skin inflammation, but as noted above, it shows that resistant bovines recruit significantly more CD3^+^ T cells to tick bite lesions than do susceptible hosts.

Predicted lipocalins were by far the most abundant protein category in predicted tick secreted proteins and were g over-represented in libraries derived from nymphs feeding on susceptible hosts relative to resistant hosts. Lipocalins bind to small molecules such as histamine, serotonin, leukotrienes and volatile odorants. Mast cells and basophils will release histamine in response to the damage inflicted by tick bites and promote inflammation and wound healing. Thus the expression profile of this category of molecules in tick salivary glands is also compatible with the patterns of inflammation seen in the two types of hosts. Functional characterization of lipocalins from hard ticks demonstrates that they can bind serotonin, histamine and leukotrienes [75–77]. Functions have been demonstrated for very few members of the large family of lipocalins (there were over one hundred coding sequences predicted to be secreted in the transcriptome of *R. microplus*) and since many lipocalins exhibit odorant-binding properties, including in arthorpods [78], it is reasonable to speculate that tick lipocalins may also bind to host odorants and thus affect the host’s semiochemicals for larvae and/or for the male ticks seeking females to mate with.

### Immunological responses of hosts to tick bites

Laceration of a host’s skin by ticks brings their mouthparts into contact with sentinel cell populations, including keratinocytes, fibroblasts, dendritic cells, mast cells, receptors that are components of the innate immune response, anti-microbial peptides, together with chemokines and cytokines involved in inflammation and wound repair [79]. The initial response to ticks suggested by this study is similar to an allergic contact dermatitis that is elicited by keratinocytes and fibroblasts producing IL-6, CXCL-8 and CCL-2 to recruit inflammatory leukocytes. This skin disease is induced by repeated skin contact with low molecular weight chemicals, known as xenobiotics or haptens, and is mediated by IL-6 and TNF-α cytokines [80]. Tick saliva is a complex xenobiotic substance that is composed of low molecualr weight proteins/polypeptides, plus lipids and carbohydrates, with an array of different functions. In the model employed in this study, heavily infested susceptible hosts can receive approximately 200 ml of saliva, containing milligrams of protein [81]. Saliva is responsible for the success of tick attachment, blood-feeding and transmission of pathogens [3, 82]. Infested skins from susceptible hosts exhibited less damage than skins from resistant hosts suffering bites from the same developmental stage and ticks re stably attached. Because of the increased tick loads, the gene expression profiles in the skin of susceptible cattle contained more enzymes involved in detoxification of tissues, these enzymes also produce an array of skin chemicals that have the potential to attract more *R. microplus* larvae.

Molecular mechanisms of feeding lesions caused by *R. microplus* have been examined comparatively in skins of resistant and susceptible cattle by other investigators [83, 84]. Skin responses have also been examined at the molecular level in mice infested with nymphs of *Ixodes scapularis* [85]. However, none of those studies examined molecular and histological aspects of host skin in parallel with transcriptional and behavioral aspects of tick. The present work followed artificial infestations in hosts of two bovine breeds that are known to be genetically resistant and susceptible to *R. microplus* and that had no previous exposure to ticks. By contrast earlier studies, by Piper and colleagues [83] and by Carvalho and colleagues [84], employed hosts that had been infested many times. An important difference between the present gene profiling study and the previous ones [83, 84] is the care taken to obtain skin samples with procedures that avoid mixing and, therefore, diluting RNA from inflamed or wounded tissue with RNA from stressed but non-inflamed tissue, thus confounding results for the molecular composition of local and systemic reactions to bites, and also importantly, compromising the validity of statistical tests that determine the significance of putative differentially expressed genes.

Recent work that examined gene expression in skin in a murine model of infestations with *I. scapularis* ticks showed that, compared with secondary infestations, innate immunity was delayed and Th17 responses inhibited in response to primary infestations with ticks. The mouse model used is in some ways comparable to the Holstein breed of cattle used in the present work, because the mouse is susceptible to ticks even after secondary infestations [85], and,although the acquired immune response is more potent after a secondary infestation, it never reaches the level of resistance shown by indicine cattle.

## Conclusions

Our observations on the cellular composition of the inflammation recruited to tick bite-associated lesions concur with the corresponding expression profiles of chemokines in skin. They also show that there are significant differences between skin from the bovine hosts presenting with different levels of resistance to tick infestations. The cellular composition of these hosts’ reactions also concurs with the expression profile of sequences encoding immunomodulatory and anti-inflammatory proteins in ticks feeding on the corresponding hosts, tick-resistant or susceptible. Furthermore, tick-resistant hosts recruit inflammatory responses earlier than susceptible hosts and with a molecular profile that is similar to that observed in allergic contact dermatitis. Differences between resistant and susceptible hosts in their expression profiles of genes encoding enzymes producing volatile compounds and differences in behavioral responses of ticks exposed to skin rubbings of resistant and susceptible hosts suggest that composition of skin semiochemicals will differ between these types of hosts. The data provides important insights into the molecular basis of differences in tick-resistant and susceptible cattle and associated modulation of the tick ‘secretome’.

## Additional files

Additional file 1: Figure S1. Experimental design and scheme for collection of tick RNA and skin samples. **a** Unfed larvae (ecloded from eggs oviposited by females fed on Holstein, 10,000 for each treatment) were kept in silk bags (previously washed in double distilled water and air dried) and rested on the neck of Nelore or Holstein bovines for 30 min in order to expose them to host odors and thenwere deposited in RNAlater prior to isolating total RNA. Another set of 10.000 larvae was released and fed on Holstein or Nelore bovines for 24 h, then were brushed off bovines and stored in RNAlater. The last set of 10,000 larvae was released on Holstein or Nelore bovines and were permitted to feed and develop into nymphs and the salivary glands were dissected from 100 nymphs and deposited in RNAlater. **b** The first set of skin biopsies was taken from Nelores (*n* = 4) and Holsteins (*n* = 4) that had never been infested with ticks; then 10,000 larvae were released on the same animals and a second set of two types of skin biopsies was taken 2 days later, one with a feeding larva in the center and one from intact skin. On the ninth day after infestation a third set of skin biopsies was taken with a feeding nymph in the center. All skin biopsies were collected with a 6 mm punch. *Abbreviations*: Bsl, baseline skin from uninfested, tick-naïve bovines; Str, stressed skin without a tick bite from bovines infested for 2 days; Lar, 2 day-feeding larva in center of biopsy; Nym, 9 day-feeding nymph in center of biopsy. (TIF 418 kb)
Additional file 2: Figure S2. Diagram of the zones where cell counts were made in tick infested skin from bovines: Zone 1: presence of an epidermal rupture, the feeding cavity matching the central bite site and the cement cone that are a big mass at the epidermis surface; zone 2: absence of cement cone and presence of inflammatory cells in the dermis surrounding the bite site; zone 3: absence of cement cone, borderline infiltration of inflammatory cells. (TIF 1740 kb)
Additional file 3: Table S1. Tick counts in Nelore and Holstein bovines for confirmation of resistance and susceptibility to infestations with *R. microplus. (DOCX 25 kb)*

Additional file 4: Table S2. Signal intensity values calculated by Robust Multi-array Average (RMA) algorithm in affy R package from Bioconductor software. Each sheet contains RMA values for samples used in a given pairwise compairison as named across the sheets. Each comparison resulted in a set of DEG presented in Table S3 (intra-breed comparisons) and Table S5 (inter-breed comparisons). *Abbreviations*: SBsl, baseline skin from susceptible bovines; RBsl, baseline skin from resistant bovines; SStr, stressed skin from susceptible bovines; RStr, stressed skin from resistant bovines; SLAr, larvae-infested skin from susceptible bovines; RLar, larvae-infested skin from resistant bovines; SNym, nymph-infested skin from susceptible bovines; RNym, nymph-infested skin from resistant bovines. (XLSX 29676 kb)
Additional file 5: Table S3. Differentially expressed genes (DEGs) in tick-infested skins (with larvae or nymph) within each breed (intra-breed comparisons) compared to stressed skin as named across the sheets. DEGs were calculated using limma R package from Bioconductor software. The up- (positive values) or downregulation (negative values) of genes are displayed in “logFC” column. *Abbreviations*: SLAr, larvae-infested skin from susceptible bovines; RLar, larvae-infested skin from resistant bovines; SNym, nymph-infested skin from susceptible bovines; RNym, nymph-infested skin from resistant bovines; SLarNym, larvae and nymph-infested skin from susceptible bovines; RLarNym, larvae and nymph-infested skin from resistant bovines. (XLSX 326 kb)
Additional file 6: Table S4. Genetic pathways and networks identified with the MetaSkin analysis software. (XLS 43 kb)
Additional file 7: Table S5. Differentially expressed genes (DEGs) between skins of different breeds (inter-breed comparisons). The types of skin (Bsl, baseline; Str, stressed; Lar, larvae-infested; Nym, nymph-infested) are given for each comparison against the susceptible breed, as named across the sheets. DEGs were calculated using limma R package from Bioconductor software. The up- (positive values) or downregulation (negative values) shown are of expression of genes from skin of the resistant breed compared to susceptible and are displayed in “logFC” column. (XLSX 232 kb)
Additional file 8: Table S6. Kinetics of attraction of larvae of R. microplus to skin chemistry from tick-resistant and tick-susceptible bovines. (DOCX 31 kb)
Additional file 9: Table S6. Total and differential leukocyte counts in skins from tick-resistant and tick-susceptible bovines (DOCX 22 kb)
Additional file 10: Figure S3. Lymphocyte phenotypes in tick-infested skins. Cryopreserved sections were stained with peroxidase-immunohistochemistry to counts local lymphocytes, as described in the methods section. The blue arrowhead marks cement cone produced by *R. microplus* showing the sections in the center of tick attachment (Zone 1 or 2, Additional file 1: Figure S1b), while black arrowhead marks the lymphocytes surrounding cement cone (original magnifications were 10× and 100×). (TIF 4394 kb)

## Abbrevations

Bsl: Bovine skin sampled before infestation (i.e. baseline skin from tick-naïve hosts)
DEG: Differentially expressed genes
FLR: Larvae derived from eggs oviposited by females fed on tick-susceptible hosts feeding for 24 h on tick-resistant hosts
FLS: Larvae derived from eggs oviposited by females fed on tick-susceptible hosts feeding for 24 h on tick-susceptible hosts
HCL: Hierarchical clustering
Lar: Bovine skin infested with a larva
log2FC: Fold change values
MeV: (Multi Experiment Viewer)
NMF: Non-negative Matrix Factorization method
Nym: Bovine skin infested with a nymph
R: Nelore, tick-resistant *Bos indicus* calves
RH: Relative humidity
RMA: Robust multiarray average
S: Holstein, tick-susceptible *Bos taurus* calves
SGNR: Salivary glands dissected from nymphs fed on tick-resistant hosts
SGNS: Salivary glands dissected from nymphs fed on tick-susceptible hosts
Str: Systemically stressed bovine skin sampled during an infestation, but without ticks
T: Temperature
ULR: Unfed larvae derived from eggs oviposited by females fed on tick-resistant hosts
ULS: Unfed larvae derived from eggs oviposited by females fed on tick-susceptible hosts
ULVR: Unfed larvae derived from eggs oviposited by females fed on tick-susceptible hosts exposed to odors of tick-resistant hosts
ULVS: Unfed larvae derived from eggs oviposited by females fed on tick-susceptible hosts exposed to odors of tick-susceptible hosts
VOC: Volatile odoriferous compounds
