# Supplementary figures and images for: Immune and biochemical responses in skin differ between bovine hosts genetically susceptible and resistant to the cattle tick Rhipicephalus microplus
Source: Parasit Vectors. 2017 Jan 31;10:51. doi: 10.1186/s13071-016-1945-z (PMC5282843; doi:10.1186/s13071-016-1945-z)

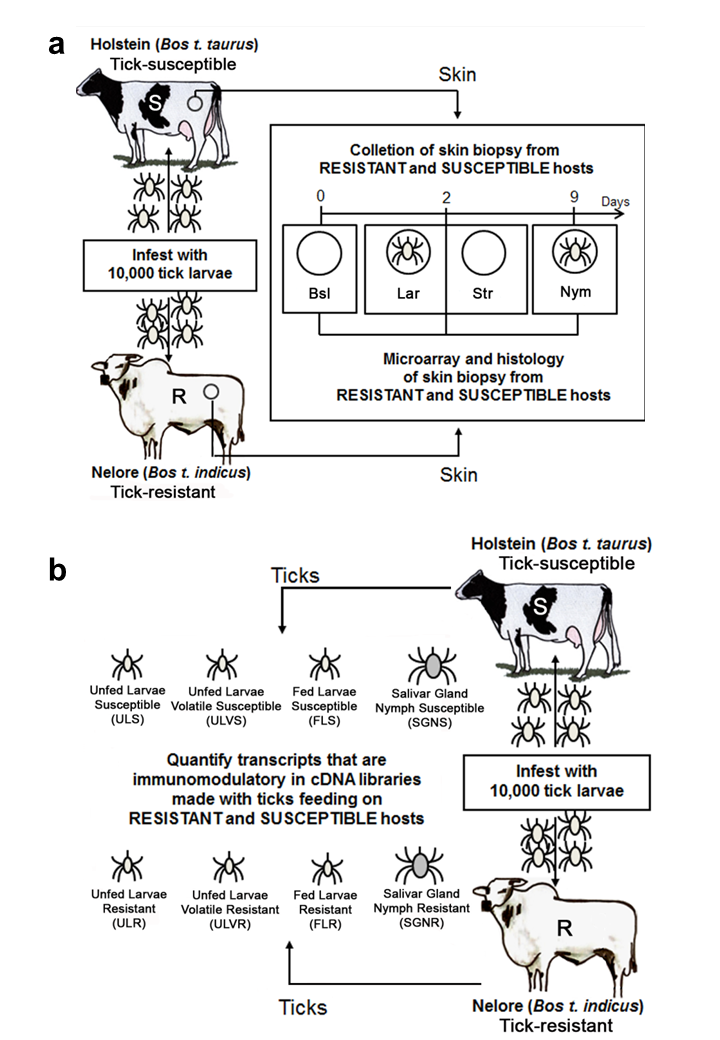

Supplement: Additional file 1: Figure S1. — Experimental design and scheme for collection of tick RNA and skin samples. a Unfed larvae (ecloded from eggs oviposited by females fed on Holstein, 10,000 for each treatment) were kept in silk bags (previously washed in double distilled water and air dried) and rested on the neck of Nelore or Holstein bovines for 30 min in order to expose them to host odors and thenwere deposited in RNAlater prior to isolating total RNA. Another set of 10.000 larvae was released and fed on Holstein or Nelore bovines for 24 h, then were brushed off bovines and stored in RNAlater. The last set of 10,000 larvae was released on Holstein or Nelore bovines and were permitted to feed and develop into nymphs and the salivary glands were dissected from 100 nymphs and deposited in RNAlater. b The first set of skin biopsies was taken from Nelores (n = 4) and Holsteins (n = 4) that had never been infested with ticks; then 10,000 larvae were released on the same animals and a second set of two types of skin biopsies was taken 2 days later, one with a feeding larva in the center and one from intact skin. On the ninth day after infestation a third set of skin biopsies was taken with a feeding nymph in the center. All skin biopsies were collected with a 6 mm punch. Abbreviations: Bsl, baseline skin from uninfested, tick-naïve bovines; Str, stressed skin without a tick bite from bovines infested for 2 days; Lar, 2 day-feeding larva in center of biopsy; Nym, 9 day-feeding nymph in center of biopsy. (TIF 418 kb) [file 13071_2016_1945_MOESM1_ESM.tif]

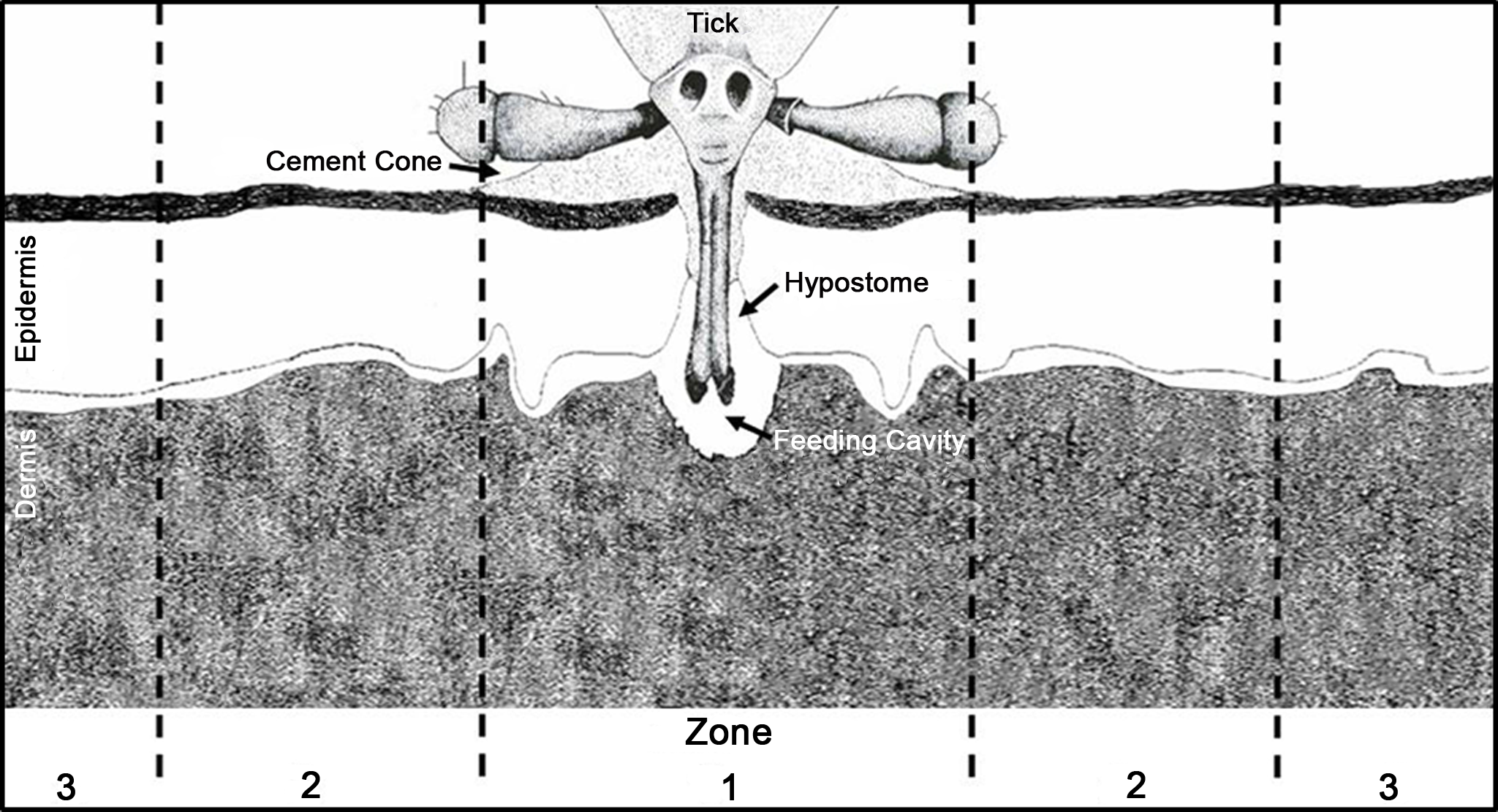

Supplement: Additional file 2: Figure S2. — Diagram of the zones where cell counts were made in tick infested skin from bovines: Zone 1: presence of an epidermal rupture, the feeding cavity matching the central bite site and the cement cone that are a big mass at the epidermis surface; zone 2: absence of cement cone and presence of inflammatory cells in the dermis surrounding the bite site; zone 3: absence of cement cone, borderline infiltration of inflammatory cells. (TIF 1740 kb) [file 13071_2016_1945_MOESM2_ESM.tif]

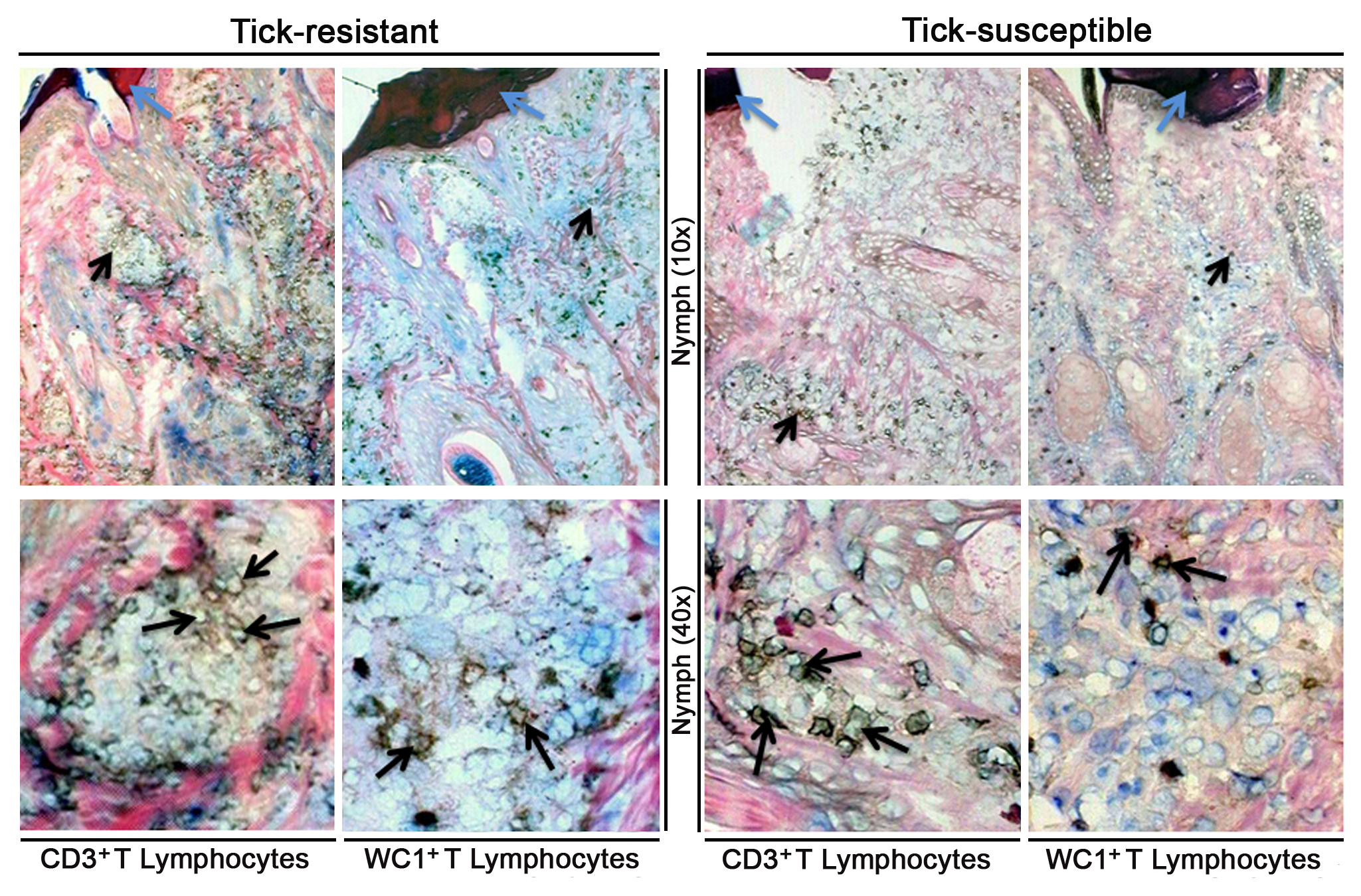

Supplement: Additional file 10: Figure S3. — Lymphocyte phenotypes in tick-infested skins. Cryopreserved sections were stained with peroxidase-immunohistochemistry to counts local lymphocytes, as described in the methods section. The blue arrowhead marks cement cone produced by R. microplus showing the sections in the center of tick attachment (Zone 1 or 2, Additional file 1: Figure S1b), while black arrowhead marks the lymphocytes surrounding cement cone (original magnifications were 10× and 100×). (TIF 4394 kb) [file 13071_2016_1945_MOESM10_ESM.tif]
