# Supplementary material for: Immune and biochemical responses in skin differ between bovine hosts genetically susceptible and resistant to the cattle tick Rhipicephalus microplus
Source: Parasit Vectors. 2017 Jan 31;10:51. doi: 10.1186/s13071-016-1945-z (PMC5282843; doi:10.1186/s13071-016-1945-z)
Supplement: Additional file 3: Table S1. — Tick counts in Nelore and Holstein bovines for confirmation of resistance and susceptibility to infestations with R. microplus. (DOCX 25 kb) [file 13071_2016_1945_MOESM3_ESM.docx]

Table S1: Tick counts in Nelore and Holstein bovines for confirmation of resistance and susceptibility to infestations with *R. microplus*

| Bovine breeds | Animal ID | Number of ticks/animal^1^ | Means/SD |
| --- | --- | --- | --- |
| NELORE  (Tick-resistant) |  |  |  |
|  | 724 | 0 |  |
|  | 727 | 76 |  |
|  | 733 | 68 |  |
|  | 734 | 26 |  |
|  |  |  | 42.5 ± 35.8* |
| HOLSTEIN  (Tick-susceptible) |  |  |  |
|  | 02 | 412 |  |
|  | 03 | 436 |  |
|  | 10 | 840 |  |
|  | 13 | 700 |  |
|  |  |  | 597.0 ± 208.0* |

^1^Values in column represent the number of female ticks larger than 4 mm counted on the left side of each experimental bovine on the 21^st^ day after release of larvae and then multiplied by two to estimate the total number of ticks per animal; ^*^Values differ significantly (*P* = 0.019, t test).
