# Supplementary material for: Immune and biochemical responses in skin differ between bovine hosts genetically susceptible and resistant to the cattle tick Rhipicephalus microplus
Source: Parasit Vectors. 2017 Jan 31;10:51. doi: 10.1186/s13071-016-1945-z (PMC5282843; doi:10.1186/s13071-016-1945-z)
Supplement: Additional file 8: Table S6. — Kinetics of attraction of larvae of R. microplus to skin chemistry from tick-resistant and tick-susceptible bovines. (DOCX 31 kb) [file 13071_2016_1945_MOESM8_ESM.docx]

Table S6: Kinetics of attraction of larvae of *R. microplus* to skin chemistry from tick-resistant and tick-susceptible bovines.

| Host | Time after release of larvae | % of area of strip containing skin chemistry occupied by larvae | Means/SD |
| --- | --- | --- | --- |
| Tick-resistant (A)^1^ |  |  |  |
|  | 5 minutes | 1.3 |  |
|  |  | 1.9 |  |
|  |  | 2.1 |  |
|  |  | 2.3 |  |
|  |  | 4.1 |  |
|  |  |  | 2.0 ± 1.5^AAA^ |
|  | 10 minutes | 6.6 |  |
|  |  | 13.1 |  |
|  |  | 15.2 |  |
|  |  | 18.6 |  |
|  |  |  | 13.4 ± 5.1^AAA*2^ |
|  | 15 minutes | 15.7 |  |
|  |  | 14.4 |  |
|  |  | 12.4 |  |
|  |  | 9.4 |  |
|  |  | 10.7 |  |
|  |  | 13.9 |  |
|  |  |  | 12.8 ± 2.4^AAA^ |
| Tick-susceptible (B) |  |  |  |
|  | 5 minutes | 1.6 |  |
|  |  | 2.3 |  |
|  |  | 3.2 |  |
|  |  | 6.8 |  |
|  |  | 9.9 |  |
|  |  | 1.6 |  |
|  |  |  | 4.8 ± 3.5^BBB^ |
|  | 10 minutes | 16.8 |  |
|  |  | 18.4 |  |
|  |  | 27.9 |  |
|  |  | 22.2 |  |
|  |  | 23.8 |  |
|  |  | 23.2 |  |
|  |  | 16.8 |  |
|  |  |  | 22.1 ± 4.0^BBB*^ |
|  | 15 minutes |  |  |
|  |  | 22.5 |  |
|  |  | 21.9 |  |
|  |  | 18.8 |  |
|  |  | 17.3 |  |
|  |  | 23.3 |  |
|  |  | 20.4 |  |
|  |  | 19.5 |  |
|  |  | 22.5 |  |
|  |  |  | 20.5 ± 2.2^BBB**^ |
| Human (C) |  |  |  |
|  | 5 minutes | 0.0 |  |
|  |  |  | 0.0 ± 0.0^ccC*^ |
|  | 10 minutes |  |  |
|  |  | 0.0 |  |
|  |  | 0.1 |  |
|  |  |  | 0.1 ± 0.7^ccC**^ |
|  | 15 minutes |  |  |
|  |  | 0.5 |  |
|  |  | 0.8 |  |
|  |  | 1.7 |  |
|  |  | 1.3 |  |
|  |  | 1.8 |  |
|  |  | 2.6 |  |
|  |  | 3.6 |  |
|  |  | 3.2 |  |
|  |  | 0.5 |  |
|  |  | 0.8 |  |
|  |  |  | 2.0 ± 1.1^ccC**^ |
| Control strip (D) |  |  |  |
|  | 5 minutes | 0.0 |  |
|  |  | 0.0 |  |
|  |  |  | 0.0 ± 0.0^ddD**^ |
|  | 10 minutes |  |  |
|  |  | 0.0 |  |
|  |  | 0.5 |  |
|  |  |  | 0.3 ± 0.4^ddD**^ |
|  | 15 minutes |  |  |
|  |  | 3.5 |  |
|  |  | 2.9 |  |
|  |  | 6.5 |  |
|  |  | 6.7 |  |
|  |  | 9.6 |  |
|  |  | 10.4 |  |
|  |  | 9.3 |  |
|  |  | 8.2 |  |
|  |  | 3.5 |  |
|  |  | 2.9 |  |
|  |  |  | 7.0 ± 2.8^ddD**^ |

^1^Values in column followed by (*) differ significantly at the level of P < 0.05 and by (**) differ significantly at the level of P < 0.001 in inter-host comparisons. ^2^Values in a column followed by capital letter differ significantly (P>0.005), intra-host comparison.
