# Supplementary material for: Immune and biochemical responses in skin differ between bovine hosts genetically susceptible and resistant to the cattle tick Rhipicephalus microplus
Source: Parasit Vectors. 2017 Jan 31;10:51. doi: 10.1186/s13071-016-1945-z (PMC5282843; doi:10.1186/s13071-016-1945-z)
Supplement: Additional file 9: Table S6. — Total and differential leukocyte counts in skins from tick-resistant and tick-susceptible bovines (DOCX 22 kb) [file 13071_2016_1945_MOESM9_ESM.docx]

Table S7: Total and differential leukocyte counts in skins from tick-resistant (R) and tick-susceptible bovines (S)

| Skin | Host | Mean cell couts ± SD | | | | | | | | |
| --- | --- | --- | --- | --- | --- | --- | --- | --- | --- | --- |
|  |  | Total cells | Mast cell | Mononuclears | Eosinophils | Basophils | Neutrophis | CD3^+^ T Lymphocytes | WC1^+^ T Lymphocytes | CD21^+^ B Lymphocytes |
| Baseline | R (A) ^1^ | 64.0 ± 15.8^aAA^ | 26.0 ± 4.5^AaA^ | 50.7 ± 10.6^Aaa^ | 0.2 ± 0.3^aaa^ | 0.0 ± 0.0^aaA^ | 0.2 ± 0.3^aaA^ | 23.1 ± 7.4^aaa^ | 12.5 ± 9.3^aaa^ | 12.1 ± 3.1^aaa^ |
|  | S (B) | 62.0 ± 9.6^BBB^ | 32.5 ± 7.6^BBB^ | 52.0 ± 15.8^BbB^ | 0.0 ± 0.0^bBb^ | 0.0 ± 0.0^bbb^ | 0.0 ± 0.0^bBb^ | 18.8 ± 4.2^bbB^ | 14.3 ± 1.7^bbb^ | 11.3 ± 0.4^bbB^ |
| Larvae | R | 320.4 ± 78.0^AAA^ | 11.3 ± 5.9^AaA*2^ | 70.7 ± 18.1^aaA^ | 14.0 ± 3.4^aaa*^ | 0.4 ± 0.5^aaa^ | 6.2 ± 4.2^aaa^ | 73.8 ± 40.5^aaa^ | 32.6 ± 17.8^aaa^ | 2.8 ± 2.5^aaa^ |
|  | S | 325.5 ± 57.5^BBb^ | 2.1 ± 2.4^BBb*^ | 77.3 ± 2.0^bbB^ | 3.3 ± 2.1^Bbb*^ | 0.7 ± 1.2^bbb^ | 5.3 ± 5.2^BBb^ | 54.9 ± 23.8^bbb^ | 19.8 ± 8.2^bbB^ | 7.7 ± 6.3^bbb^ |
| Nymph | R | 320.2 ± 209.6^AAA^ | 2.0 ± 2.4^AAA^ | 54.9 ± 5.0^AAA^ | 5.3 ± 4.2^aaa*^ | 0.2 ± 0.3^AAa*^ | 34.0 ± 18.8^Aaa^ | 138.6 ± 49.2^aAa*^ | 30.1 ± 30.9^aaa*^ | 39.9 ± 39.7^aaa^ |
|  | S | 525.8 ± 82.3^Bbb^ | 3.5 ± 3.3^BBb^ | 74.4 ± 7.6^BBB^ | 28.0 ± 10.7^Bbb*^ | 2.9 ± 1.3^bbb*^ | 6.9 ± 3.2^bbb^ | 60.9 ± 17.3^Bbb*^ | 5.2 ± 3.6^bBb*^ | 2.3 ± 1.9^Bbb^ |
| Stressed | R | 185.7 ± 33.4 ^aAA^ | 13.0 ± 2.2^AaA*^ | 83.4 ± 2.7 ^AaA^ | 9.8 ± 2.9^aaa**^ | 0.0 ± 0.0^aaa^ | 0.1 ± 0.2 ^aaa^ | 32.9 ± 9.2^aaA^ | 22.6 ± 7.8^aaa^ | 12.7 ± 3.1^aAa^ |
|  | S | 173.0 ± 20.2^BBb^ | 7.1 ± 1.9^BBB*^ | 83.6 ± 4.2^BBB^ | 3.0 ± 1.5^bbB**^ | 0.0 ± 0.0^bbb^ | 0.1 ± 0.3 ^bBb^ | 36.2 ± 13.5^bbb^ | 16.7 ± 6.2^bbb^ | 12.3 ± 3.6^bbb^ |

^1^Values in a column followed by (*) differ significantly (P < 0.05) and (**) differ significantly (P = < 0.001), inter-breed comparison. ^2^Values in a column followed by capital letter differ significantly (P < 0.05), intra-breed comparison.
